# Bioinspired Claw‐Engaged Adhesive Microparticles Armed with γGC Alleviate Ulcerative Colitis via Targeted Suppression of Macrophage Ferroptosis

**DOI:** 10.1002/advs.202503903

**Published:** 2025-04-29

**Authors:** Rong Wang, Jianwei Zhu, Jinyi Zhou, Jinyang Li, Min Wang, Yuqi Wu, Danshan Zhao, Xiancheng Chen, Xiaoyuan Chen, Yuetong Wang, Jianhua Zou

**Affiliations:** ^1^ Jiangsu Province Key Laboratory for Molecular and Medical Biotechnology College of Life Science Nanjing Normal University Nanjing Jiangsu 210046 China; ^2^ Hunan Provincial Key Laboratory of the Research and Development of Novel Pharmaceutical Preparations Changsha Medical University Changsha Hunan 410219 China; ^3^ Departments of Diagnostic Radiology Surgery Chemical and Biomolecular Engineering and Biomedical Engineering Yong Loo Lin School of Medicine and College of Design and Engineering National University of Singapore Singapore 119074 Singapore; ^4^ Hepatobiliary Center The First Affiliated Hospital of Nanjing Medical University Nanjing Jiangsu 210029 China; ^5^ School of Food Science and Pharmaceutical Engineering Nanjing Normal University Nanjing Jiangsu 210046 China; ^6^ Department of Critical Care Medicine Nanjing Drum Tower Hospital The Affiliated Hospital of Nanjing University Medical School Nanjing Jiangsu 210029 China

**Keywords:** ferroptosis, M1 reprogramming, PI3K/AKT pathway, ulcerative colitis

## Abstract

Ulcerative colitis (UC) is a chronic inflammatory bowel disease, characterized by focal iron overload. Herein, we reported that γ‐glutamylcysteine (γGC) deletion in UC lesions intensified the disease by depleting intracellular GSH to induce macrophage ferroptosis, leading to macrophage M1 reprogramming and eventually exacerbating inflammation. To counteract this, the advanced microparticles (MPs)‐based delivery system is selected to encapsulate γGC. The resulting γGC‐MPs displayed the same porous and spiky morphology as their substrate's natural pollens, resulting in improved intestinal adhesion and enhanced lesion contact of γGC‐MPs. Our results demonstrated that exogenous γGC supplementation could inhibit macrophage M1 polarization by restraining ferroptosis, as well as suppressing the PI3K/AKT pathway and TNF signaling pathway. Compared with free γGC, γGC‐MPs significantly alleviated typical UC symptoms in dextran sulfate sodium (DSS)‐induced colitis, evidenced by reduced intestinal inflammation, restored intestinal barrier function, and improved microbiota composition. Consequently, this study addressed critical gaps in understanding the causes of ferroptosis and its impact on macrophage reprogramming in UC, offering a novel synergistic therapeutic strategy for UC.

## Graphical abstract

γGC deficiency in ulcerative colitis drives macrophage ferroptosis‐mediated inflammation. Claw‐engaged microparticles armed with γGC (γGC‐MPs) enhance lesion targeting, promoting localized γGC release and tissue uptake, thereby inhibiting macrophage ferroptosis. On the cover, Monkey King (Macrophage) armed with Golden Cudgel (Ferroptosis) was bound by the Immortal‐Binding Rope and subsequently sealed beneath the Five‐Fingered Mountain (γGC‐MPs) to prevent his further mischief.

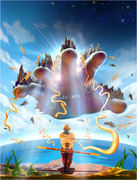

## 1 Introduction

Ulcerative colitis (UC) is a complex and long‐lasting inflammatory bowel disease marked by persistent and recurring inflammation affecting the colon and rectum, and its exact pathogenesis remains unclear.^[^ ^1^ ^]^ Current options against UC in the clinic, especially anti‐inflammatory treatments, exhibit limited therapeutic effectiveness and have inevitable drawbacks, including severe long‐term side effects and the development of anti‐drug antibodies,^[^ ^2^, ^3^ ^]^ seriously influencing the prognosis of UC patients. Although the onset of UC is still elusive, emerging evidence indicates that UC is primarily driven by hyperactive immune responses, accompanied by intestinal mucosal barrier dysfunction and disturbances in the gut microbiota.^[^ ^4^, ^5^, ^6^ ^]^ Macrophages, crucial components of the innate immune system, are key regulators of intestinal microenvironment homeostasis.^[^ ^7^, ^8^ ^]^ They exhibit remarkable phenotypic flexibility, involving classically activated pro‐inflammatory macrophages (M1‐like phenotype) and alternatively activated anti‐inflammatory macrophages (M2‐like phenotype), adapting to specific spatial and temporal pathophysiological environments.^[^ ^9^ ^]^ However, the function of macrophages playing in intestinal inflammation is like a “double‐edged sword”. Under normal conditions, macrophages protect the intestine from inflammatory damage. While for UC patients, they potentially destabilize the intestinal microenvironment, and the disorder of intestinal inflammation is also tied to the change of macrophage polarization. Tight regulation is thereby necessary to prevent excessive tissue damage.^[^ ^10^ ^]^ Given this type of reprogramming flexibility shown by macrophages, regulatory strategies targeting them are undoubtedly promising for alleviating UC symptoms.

Cell death in inflamed areas undermines the intestinal barrier and worsens the inflammatory response.^[^ ^11^, ^12^ ^]^ Ferroptosis, a newly recognized type of regulatory cell death, is initiated by iron overaccumulation, depletion of glutathione (GSH), inactivation of glutathione peroxidase 4 (GPX4), overexpression of abstract long‐chain acyl‐coenzyme A (CoA) synthase 4 (ACSL4), and lipid peroxidation,^[^ ^13^ ^]^ notably characterized by iron deposition, abnormal lipid peroxidation. Existing research has revealed that ferroptosis is tightly associated with the occurrence of UC.^[^ ^14^ ^]^ It promotes the release of inflammatory factors in macrophages by activating inflammatory signaling pathways, aggravating the condition of UC. The initiation of ferroptosis in macrophages also threatens intestinal epithelial cells, increasing the permeability of the intestinal, and destroying the intestinal barrier function, thereby exacerbating inflammation and tissue damage. Therefore, paying attention to relevant regulatory nodes of ferroptosis in macrophages may inspire the exploration of efficient therapeutic modalities for UC.

Serving as an indispensable antioxidant, GSH maintains intracellular redox balance by scavenging free radicals and peroxides.^[^ ^15^ ^]^ GSH depletion is considered as one of the inducers of ferroptosis, leading to UC symptoms via further oxidative stress.^[^ ^16^ ^]^ Restoring GSH levels in macrophages is thus of great significance in inhibiting ferroptosis and treating UC. Supplying GSH was initially selected as a direct candidate to alleviate GSH depletion. Unfortunately, the uptake of extracellular GSH is seriously restricted due to the lack of the membrane‐bound ectoenzyme γ‐glutamyltranspeptidase as well as the presence of an unfavorable concentration gradient. Meanwhile, its de novo synthesis is also limited by two critical rate‐limiting steps: the conversion of glutamate and cysteine into γ‐glutamylcysteine (γGC) through the action of γ‐glutamylcysteine ligase, and subsequently the production of GSH from γGC and glycine, which is facilitated by glutathione synthetase.^[^ ^17^ ^]^ Alternative therapeutical is highly desired to restore intracellular GSH levels. While *N*‐acetyl‐*l*‐cysteine (NAC) has been applied in the treatment of sepsis as either a direct substitute for GSH or a precursor for its synthesis, its effectiveness in clinical settings is still debated.^[^ ^18^ ^]^ γGC, a direct precursor of GSH, bypassing the thermodynamic obstacles associated with transmembrane transport, and being readily absorbed by a variety of cells to facilitate GSH synthesis,^[^ ^19^ ^]^ is thus to be recognized as one prospective target in UC treatment.

Current approaches targeting and reprogramming macrophages to treat UC remain in their infancy. Adopting advanced drug delivery systems may drive its progress. Pollen grains, particularly those from sunflowers, are ideal natural carriers for the targeted delivery of drugs because they are hollow and porous microparticles (MPs), which are conducive to drug loading. In addition, their exquisite spiky structure increases the specific surface area, thereby providing more molecular adsorption sites and enhancing tissue adhesion, especially in the inflammatory regions of the colon and rectum.^[^ ^20^, ^21^ ^]^ Inspired by this point, a natural material‐based delivery system with enhanced surface adhesion and sustained drug release capacities has been developed, modulating the polarization of macrophages, fighting against oxidative stress, as well as suppressing ferroptosis and inflammation, to alleviate UC symptoms and promote the intestinal barrier repair.

**Figure 1:**
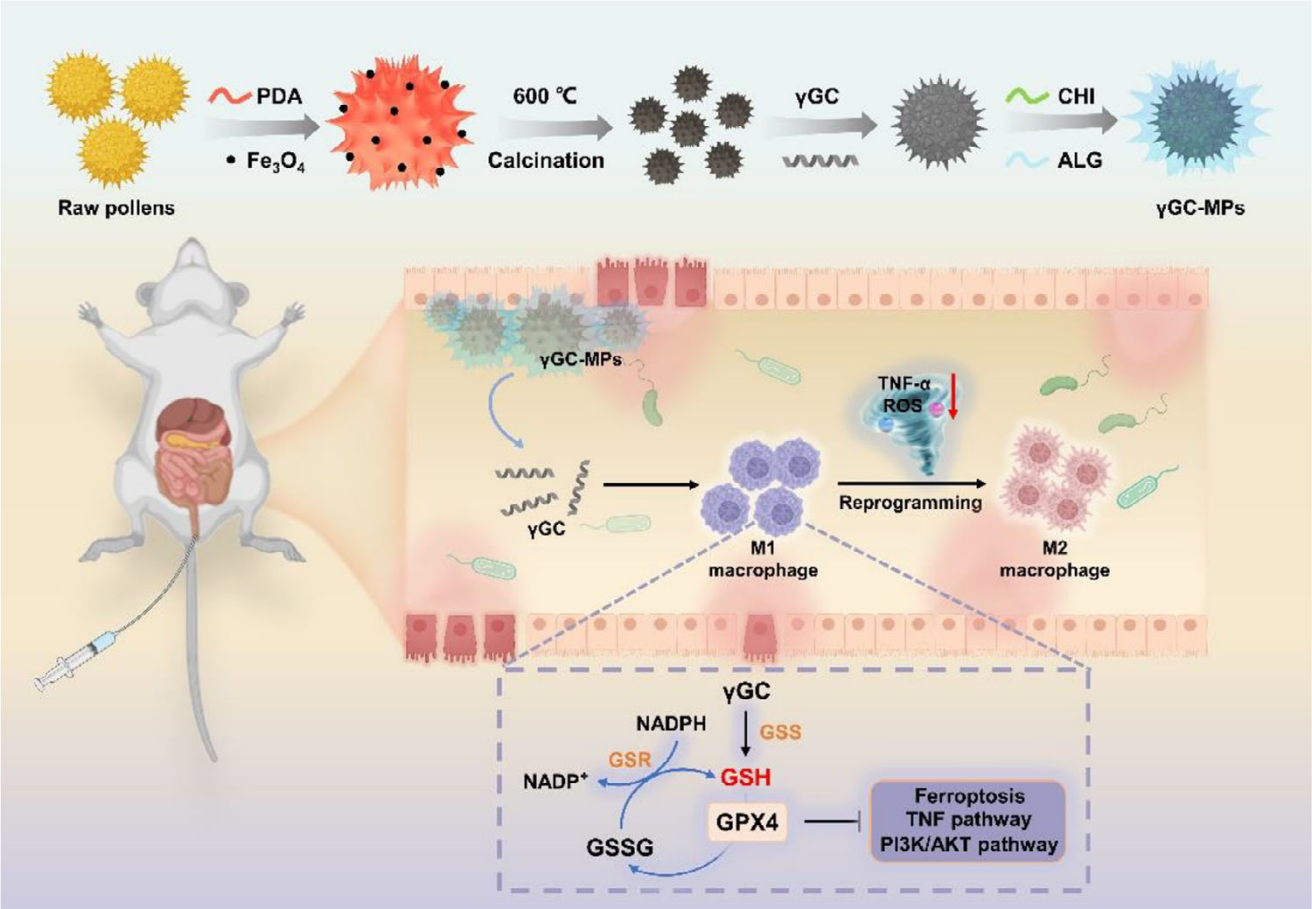
Schematic illustration of γGC‐MPs construction and its detailed mechanisms for UC treatment.

In this study (**Figure** **1**), the presence of ferroptosis in lesion areas of UC patients was identified at first. The result of metabolomics analysis suggested UC progression was tightly tied with γGC deficiency‐mediated M1 macrophage reprogramming. We thereby hypothesized that delivering γGC to the lesion regions in UC could modulate macrophage rewiring by inhibiting ferroptosis, regulating the TNF signaling pathway, and PI3K/AKT pathway to relieve UC progression. Under this hypothesis, a novel MPs delivery system, γGC‐MPs, was constructed by encapsulating γGC into calcinated Fe_3_O_4_ magnetic nanoparticles (NPs)‐coated pollens, further decorated with chitosan (CHI) and alginate (ALG). The resulting MPs exhibited porous and spiky morphology, with the retention time in the inflammatory sites as long as 72 h to accomplish the targeted release of γGC. Subsequent studies confirmed the role of γGC‐MPs played in remodeling macrophages and suppressing ferroptosis in vitro and in vivo. Furthermore, in dextran sulfate sodium (DSS)‐induced acute colitis mice, the administration of γGC‐MPs obviously relieved UC symptoms and optimized the gut microbiome, indicating the therapeutical potential of γGC‐MPs in UC and other inflammatory diseases.

## 2 Results

### 2.1 γGC Deletion‐Mediated Ferroptosis Susceptibility Contributed to Macrophage Reprogramming in Human UC

**Figure 2:**
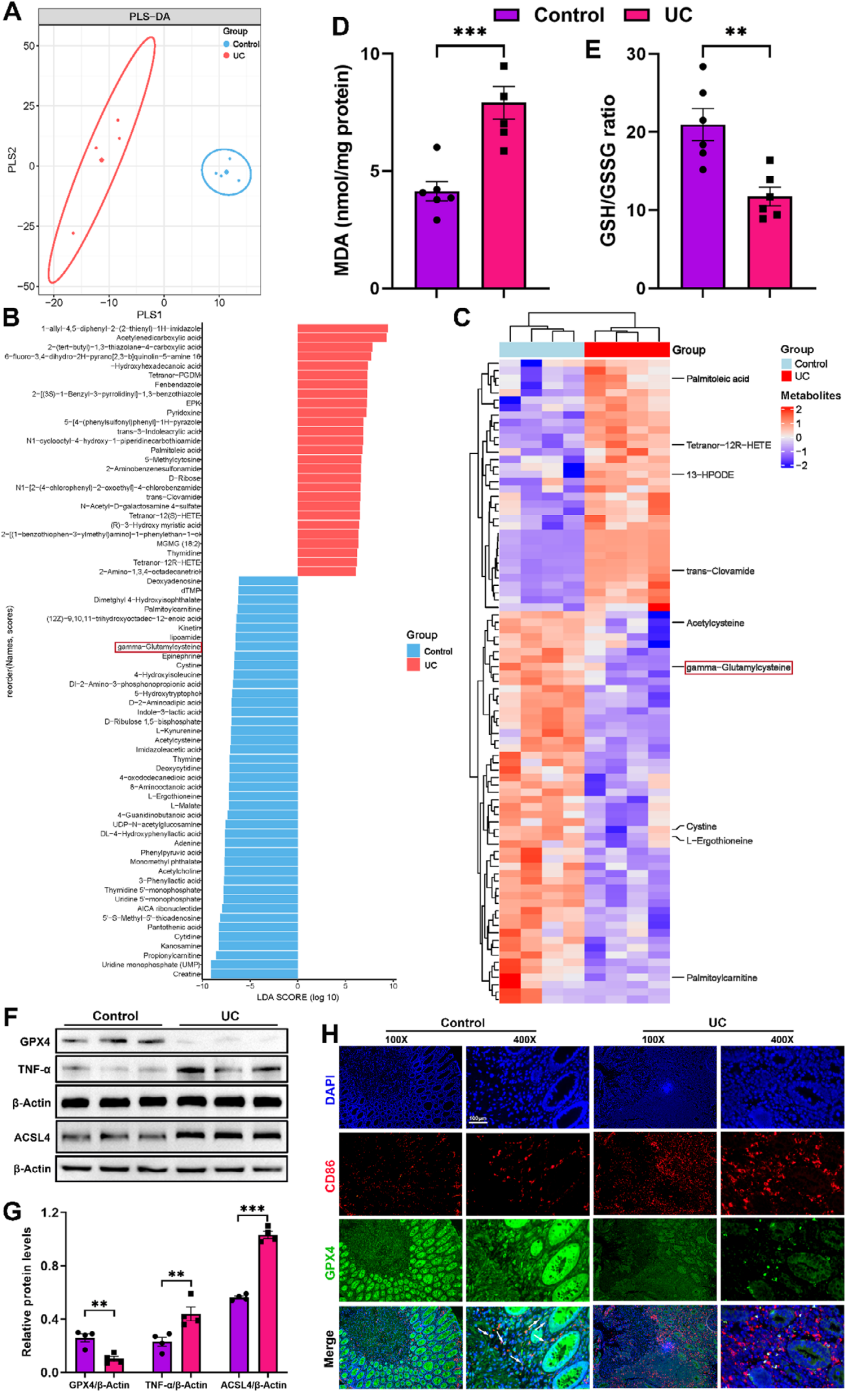
The deletion of γGC and its role in ferroptosis is related to the reprogramming of macrophages in human UC. A) The profile and differences of the metabolites analyzed by PLS‐DA. B,C) Significantly differential metabolites were identified between endoscopic colon tissue samples of UC patients and healthy volunteers (|LDA score| > 4 and *P* < 0.05). D,E) The levels of MDA and GSH/GSSG in endoscopic colon tissues of UC patients and healthy volunteers. F,G) Expressions of GPX4, ACSL4, and TNF‐α in endoscopic colon tissues were examined by immunoblot. H) Dual IF staining of CD86 and GPX4 in endoscopic colon tissues. Data are mean ± SEM. *P < 0.05, **P < 0.01, and ***P < 0.001, ns = no significant.

Iron overload during colon metabolism has been reported to facilitate focal inflammation.^[^ ^22^ ^]^ To verify whether iron imbalance was a key factor bridging colonic metabolic dysfunction to UC, untargeted metabolomics analysis of endoscopic tissue samples from UC patients and healthy volunteers was conducted at first. The profile and differences of the metabolites were analyzed by PLS‐DA following indicated groups (UC versus Control, **Figure** **2**A). There were nine significantly differential metabolites closely related to ferroptosis (|LDA score| > 4 and *P* < 0.05). Among them, γGC, the immediate precursor of GSH, showed a markedly down‐regulated trend in the tissues of UC patients, while the deficiency of GSH is one of the directly characterized features in the occurrence of ferroptosis (Figure 2B,C). We thereby evaluated the levels of GSH and lipid peroxidation (another characteristic of ferroptosis) produced MDA in the tissues of both UC patients and healthy people. The results indicated that UC patients had obviously elevated MDA concentration and evidently reduced GSH contents, compared to the control group (Figure 2,E). Further examinations of clinical samples by western blot revealed that GPX4 was markedly downregulated and ACSL4 was significantly up‐regulated, confirming the existence of ferroptosis in the UC group (Figure 2F,G). Meanwhile, the progressive expression of inflammatory marker tumor necrosis factor α (TNF‐α) was found in UC focal sites, suggesting that inflammation may be induced by the excessive ferroptosis in UC.

Macrophages display significant metabolic flexibility, and these metabolic alterations play a crucial role in driving the progression of UC.^[^ ^7^ ^]^ To investigate the impact of heterogeneous ferroptosis susceptibility on macrophage reprogramming, dual immunofluorescence (IF) staining was applied in endoscopic colon tissue samples of UC patients. The data revealed there was a negative correlation between GPX4 and CD86 expression in gut macrophages, indicating the existence of substantial M1 macrophage infiltration in the colon tissues of UC patients (Figure 2H). Combining, we speculated that γGC deletion‐mediated ferroptosis may be indispensable in inducing gut macrophage M1 polarization, further driving UC development.

### 2.2 Exogenous Supplementation of γGC Markedly Inhibited Macrophage M1 Polarization via Regulating PI3K/AKT Pathway and Inhibiting Ferroptosis

**Figure 3:**
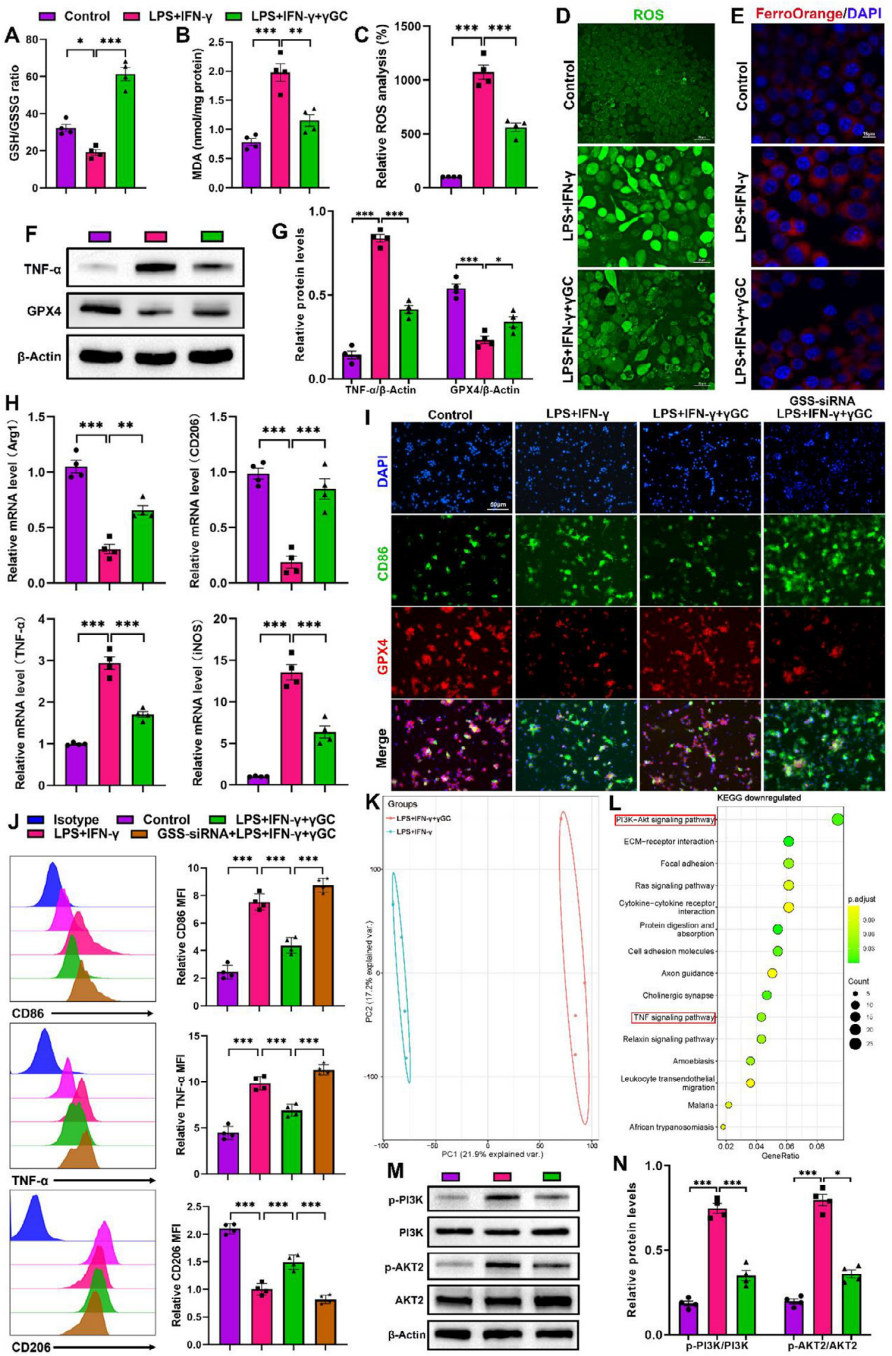
Exogenous γGC inhibited macrophage M1 polarization by suppressing ferroptosis and the PI3K/AKT pathway. A,B) The levels of GSH/GSSG and MDA in LPS and IFN‐γ‐stimulated THP‐1 cells with different treatments. C,D) Relative ROS analysis and representative images of intracellular ROS generation of LPS and IFN‐γ‐stimulated THP‐1 cells (scale bar: 1630 25 µm). E) Representative images of Fe^2+^ levels visualized by FerroOrange in LPS and IFN‐γ‐stimulated THP‐1 cells (scale bar: 15 µm). F,G) Expressions of GPX4 and TNF‐α in THP‐1 cells with different treatments were examined by immunoblot. H) mRNA expression of Arg‐1, CD206, TNF‐α, and iNOS. I) Dual IF staining of CD86 and GPX4 in THP‐1 cells (scale bar: 50 µm). J) Expressions of CD86, TNF‐α, and CD206 in THP‐1 cells were assessed by flow cytometry. K,L) Principal component analysis (PCA) and KEGG enrichment analysis of the transcriptome of THP‐1 cells with different treatments. M,N) Protein expressions of p‐PI3K, PI3K, p‐AKT2, and AKT2 were examined by immunoblot. Data are mean ± SEM. *P < 0.05, **P < 0.01, and ***P < 0.001, ns = no significant.

γGC is a direct precursor of GSH. Distinct from GSH, it does not face thermodynamic constraints for transport across membranes and is thereby easily absorbed by a range of cell types, exerting roles in restoring intracellular GSH levels, anti‐inflammation, and antioxidation. Considering the above‐mentioned speculation, we proposed that the exogenous supplementation of γGC might be a viable modality to inhibit ferroptosis of macrophages in colitis. The therapeutic capacity of γGC in vitro for macrophage was evaluated using THP‐1 cells stimulated by LPS and IFN‐γ. The morphological observations and cell viability assay showed negligible cytotoxicity of THP‐1 cells treated with γGC even up to 200 µm (Figure S1, Supporting Information). γGC treatment significantly increased GSH production while reducing MDA content and ROS levels in LPS and IFN‐γ‐stimulated THP‐1 cells (**Figure** **3**A–D). In addition, red signals decreased in the FerroOrange (a fluorescent iron probe) channel, suggesting cells treated with γGC were profoundly depleted of intracellular unstable Fe^2+^, which was conducive to the suppression of ferroptosis (Figure 3E). Subsequent immunoblot assessments revealed a notable rise in GPX4 expression accompanied by a reduction in TNF‐α levels in THP‐1 cells following treatment with γGC (Figure 3F,G). Collectively, these data demonstrated that γGC treatment augmented the efficacy of THP‐1 cells in inhibiting ferroptosis and combating inflammation and oxidative stress.

Next, the influence of γGC supplementation on macrophage polarization was evaluated by RT‐qPCR (Figure 3H). The findings indicated that the addition of exogenous γGC led to an upregulation of M2 markers (Arg‐1 and CD206) while downregulating M1 markers (TNF‐α and iNOS). The consequence of following flow cytometry was consistent with it (Figure 3J). To understand the effect of γGC on heterogeneous ferroptosis susceptibility of M1/M2 subsets, dual IF staining was applied to analyze the expressions of CD86 and GPX4 in THP‐1 cells with different treatments (Figure 3I). The results revealed that γGC hampered the occurrence of ferroptosis via inducing GPX4 expression, resulting in a reduced M1 phenotype ratio, along with an increased M2 subtype percentage, which affirmed that distinct macrophage subtypes did exhibit varying sensitivity to ferroptosis in an inflammatory environment.

Since GSH is synthesized through GSS‐catalyzed conversion of γGC, GSS siRNA was then utilized to block the production of GSH for assessing the efficacy of γGC in this process. The results showed that, compared to cells treated with LPS and IFN‐γ, the addition of GSS siRNA exacerbated GPX4 downregulation and M1 polarization, even in the presence of γGC (Figure 3I,J), indicating that the anti‐ferroptosis effect of γGC should be attributed to its ability to restore intracellular GSH levels. Consistent with these findings, we carried out rescue experiments using ferroptosis agonists RSL3 in THP‐1 cells and found that γGC treatment could effectively increase the expression of GPX4 and inhibit macrophages M1 polarization (Figure S2, Supporting Information), which strengthened the conclusion that γGC exerted therapeutic effects primarily via ferroptosis suppression.

In order to explore the underlying mechanisms of γGC exerting in THP‐1 cells, RNA sequencing analysis was conducted to identify differential genes (Figure S3, Supporting Information). Figure 3K,L revealed the most significantly altered genes were enriched onto the PI3K/AKT pathway, and the TNF signaling pathway also had substantial downregulated genes. A previous study from Arranz et al. reported that AKT serine/threonine kinase 2 (AKT2) deficiency led to macrophage polarizing to the M2 phenotype, and AKT2^−/−^ mice exhibited elevated resistance to DSS‐induced colitis, compared to wild‐type mice.^[^ ^23^ ^]^ Consistent with these findings, immunoblot analysis data demonstrated that γGC treatment effectively inhibited the macrophage polarization to M1 phenotype by repressing the phosphorylation of Phosphoinositide 3‐kinase (PI3K) and AKT2 (Figure 3M,N). Taken together, all these findings supported that γGC served as the main modulator in hindering macrophage polarizing to the M1 subtype under the inflammatory milieu by regulating the PI3K/AKT pathway and restraining ferroptosis.

### 2.3 Preparation and Characterization of γGC‐MPs

Making use of specific delivery systems to accomplish the focal release of γGC would eliminate obstacles encountered during the in vivo administration, prolonging its circulation time, improving bioavailability, and preventing drug resistance. Natural sunflower (*Helianthus annuus*) pollen grains are a type of natural material, with outstanding physical and chemical properties, such as size uniformity, excellent mechanical and chemical stability, as well as superior biocompatibility. Their primary porous morphology is conducive to drug loading, and the exquisite spiky structure increases the specific surface area, thereby providing more molecular adsorption sites and enhancing their tissue adhesion capacities (Figure S4A,B, Supporting Information). Abundant carboxyl and hydroxyl groups existing on the surface made them easy to be functionalized or formed into composite materials. Moreover, pollens possess huge reserves in nature with low collection costs, were thus chosen as the base substrates to construct microcarriers for the targeted delivery of γGC to inflammatory sites.

**Figure 4:**
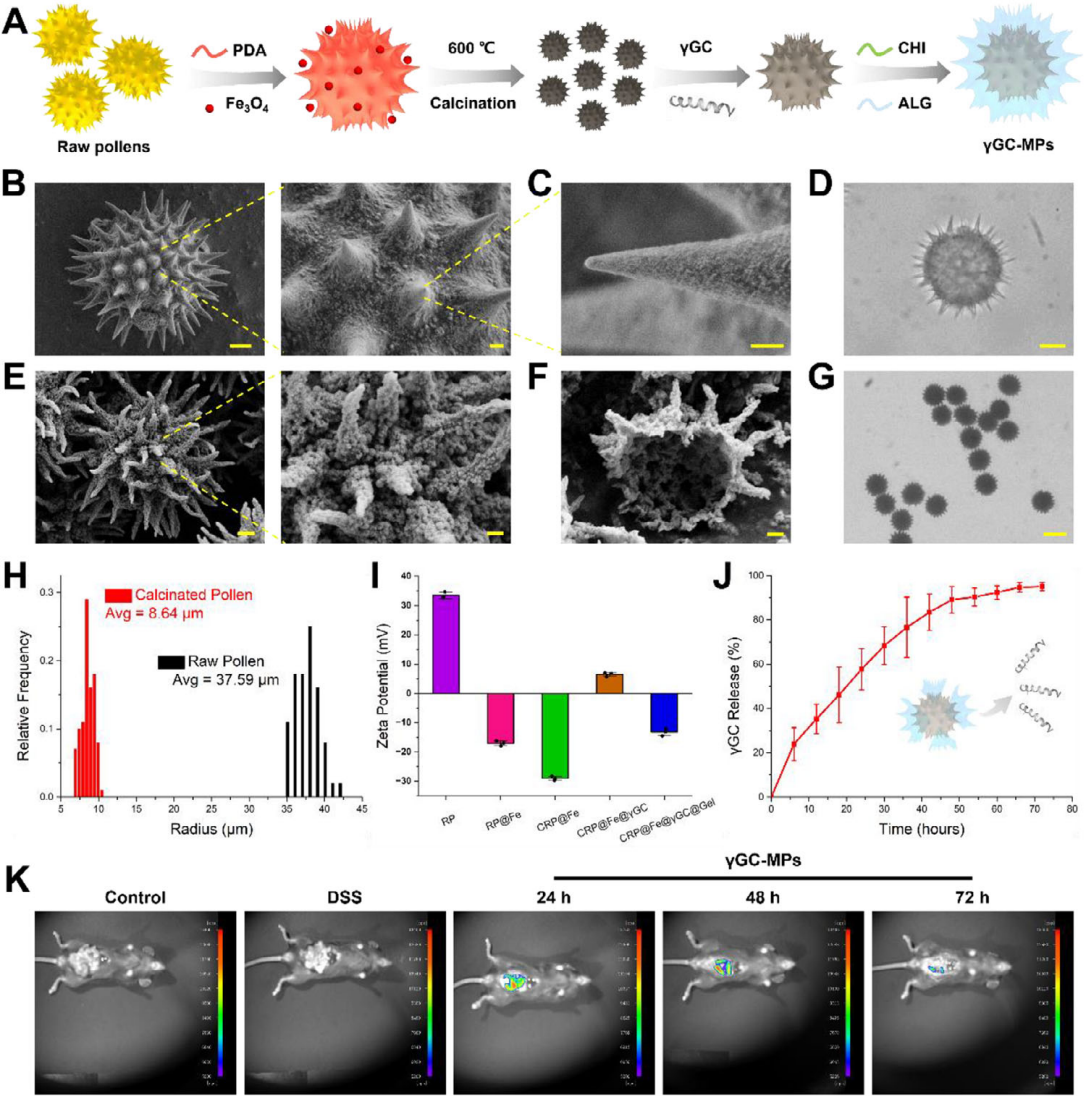
Fabrication and characterization of γGC‐MPs. A) Schematic preparation process of γGC‐MPs. B) SEM images of raw pollens decorated with Fe_3_O_4_ NPs (RP@Fe) and its magnified image (scale bars: 5 µm, left; 1 µm, right). C) Microstructure of the individual thorn of RP@Fe and the adsorption of magnetic NPs on the surface (scale bar: 1 µm). D) Optical microscopy image of RP@Fe MPs (the scale bars: 10 µm). E) Miniaturized echinate particles (CRP@Fe) obtained after calcination and the zoom‐in SEM image (scale bars: 1 µm, left; 300 nm, right). F) The internal central cavity and highly porous surface structure of CRP@Fe particles (scale bar: 1 µm). G) Optical microscopy image of CRP@Fe MPs (scale bar: 8 µm). H) Size distributions of raw pollens (RP) and calcined CRP@Fe. I) Zeta potential of five different particles (*n *= 3, CRP@Fe@γGC@Gel particles represent the resultant pollen γGC‐MPs encapsulated with active drug γGC and then functionalized with chitosan and alginate coatings). J) The cumulative release curves of γGC MPs (*n* = 3). K) In vivo fluorescence images of mice after different treatments.

An innovative prickly drug‐loaded MP was designed and fabricated, as depicted in **Figure** **4**A. In virtue of adhesion the characteristics of polydopamine (PDA), the Fe_3_O_4_ magnetic NPs were uniformly coated onto the surface of dispersed raw pollen grains to endow with magnetic response properties. The uniform distribution of NPs can be observed in SEM images (Figure 4B,C), which was also proved by the change of zeta potential value from +33.5 to −17 mV. Since pollen grains contain a variety of allergenic proteins and complex high‐molecular organic ingredients, they can trigger severe immune responses in humans. To eliminate this interference, a 600 °C calcination procedure was incorporated to carbonize the organic components of the pollens, leaving the inorganic CRP@Fe framework. Post‐calcination, the average diameter of the pollen was reduced from 37.59 to 8.64 µm, while the spikes remained intact and were further strengthened (Figure 4D–G). As shown in Figure 4F, CRP@Fe MPs had a distinct porous structure and a large central cavity, which was beneficial for the subsequent γGC encapsulation. Besides, as mentioned above, pollen possesses favorable size uniformity and the distribution of spiny structures on the surface is also relatively uniform. The particle size data and morphological consistency of the particles before and after calcination also prove this (Figure 4H, Table S6, Supporting Information). Therefore, the slight differences across batches will not affect the accuracy of subsequent drug encapsulation and release. Following that, chitosan (CHI) and alginate (ALG) were decorated onto the outer layer of CRP@Fe@γGC particles to avoid drug leakage, and the γGC‐MPs were finally obtained after the surface modification. Upon adding the gel shell, MPs’ zeta potential stabilized at −13.2 mV (Figure 4I). Furthermore, the test results of XPS, XRD, and FT‐IR confirmed the successful adhesion of Fe_3_O_4_ particles onto the original raw pollens (RPs) by PDA, as well as the successful loading of γGC and CHI/ALG (Figure S4C–E, Supporting Information). After calcination at 600 °C, most of the organic matter in RPs was removed.

Particularly worth mentioning was that both CHI/ALG and PDA/Fe_3_O_4_ layers could be successively degraded under physiological conditions to achieve the controlled release of γGC at the inflammatory site. The release kinetics of γGC from γGC‐MPs were then studied. Under the simulated acidic environment of artificial intestinal fluid, the CHI/ALG shell exhibited pH‐dependent swelling properties. The cumulative drug release curve displayed an obviously increasing trend within the initial 6 h, attributed to the rapid disintegration of the outer gel layer. Subsequently, the multi‐level pore structure of the pollen‐based MPs enabled progressive drug diffusion, explaining the 6–72 h of sustained release observed in the in vitro experiment (Figure 4J).

Based on this data, we further explore the intestinal retention time of γGC‐MPs in colitis mice. The fluorescent signals of γGC‐MPs remained in the inflammatory site for up to 72 h, implying that RPs’ spiny morphology significantly enhanced mucosal adhesion, and prolonged intestinal retention time in vivo (Figure 4K).

### 2.4 γGC‐MPs Showed Potency of Remodeling Macrophages and Anti‐Ferroptosis In Vitro

**Figure 5:**
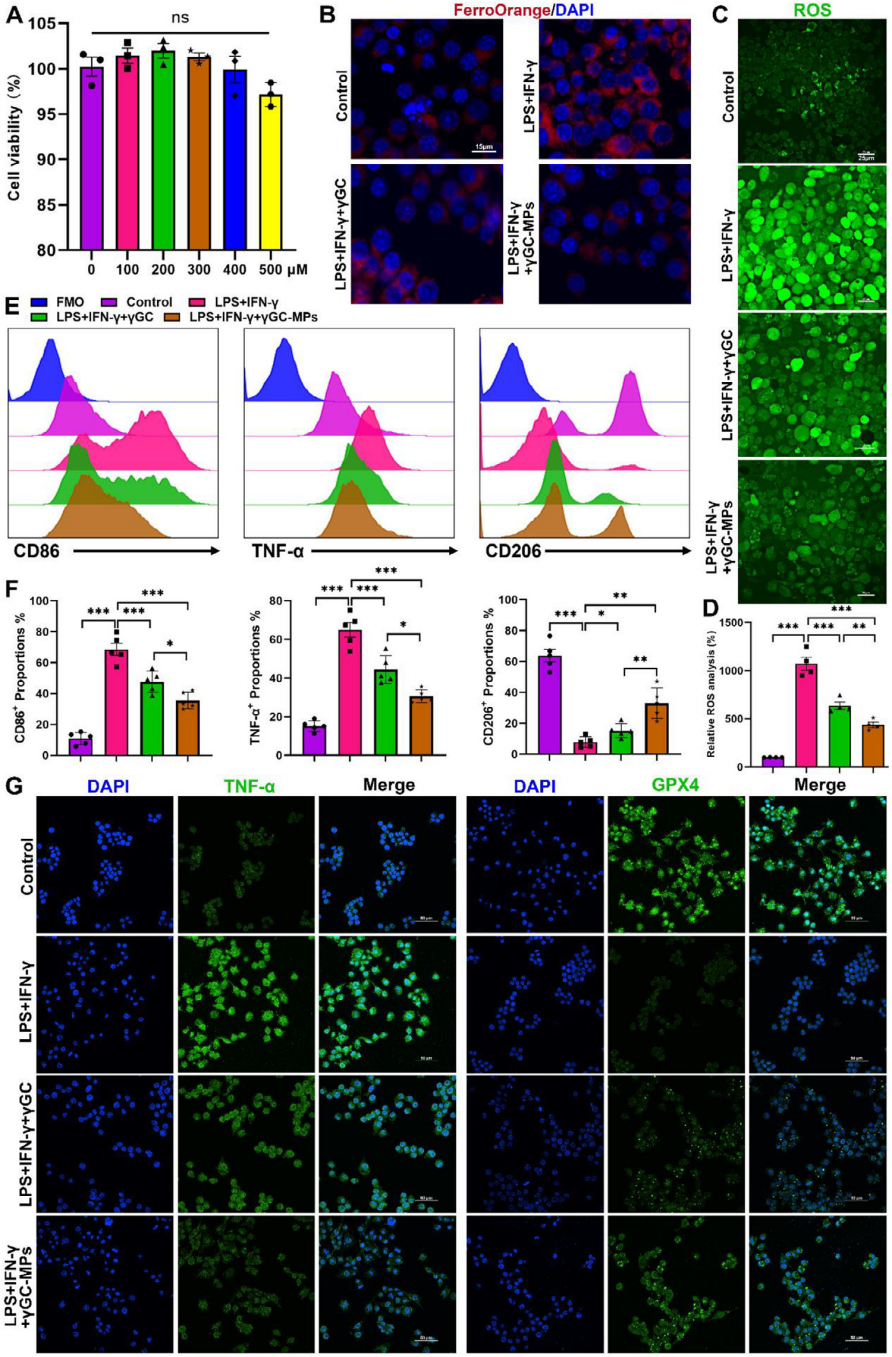
γGC‐MPs exerted robust potency in anti‐ferroptosis and reshaping macrophages and in vitro. A) Viability of THP‐1 cells treated with different concentrations of γGC‐MPs. B) Representative fluorescent images of FerroOrange stained THP‐1 cells after various treatments (scale bar: 15 µm). C,D) Representative fluorescent images of intracellular ROS levels in THP‐1 cells after different treatments (scale bar: 25 µm). E,F) Expressions of CD86, TNF‐α, and CD206 of THP‐1 cells after treatments were assessed by flow cytometry. G) ICC staining of TNF‐α and GPX4 in THP‐1 cells with distinct treatments (the scale bar: 50 µm). Data are mean ± SEM. *P < 0.05, **P < 0.01, and ***P < 0.001, ns = no significant.

To assess whether the efficacy of γGC‐MPs was different from that caused by γGC, a series of tests were conducted in THP‐1 cells. The cytotoxicity of γGC‐MPs was first evaluated in THP‐1 cells via the CCK‐8 assay (**Figure** **5**A). Even at a concentration of 500 µm, γGC‐MPs displayed negligible toxicity, indicating its excellent biocompatibility and the potential for biological applications. 200 µm was selected in subsequent experiments for a fair comparison between γGC‐MPs and γGC in vitro. Excessive Fe^2+^ can be extremely harmful because it produces oxygen radicals through the Fenton reaction, leading to irreversible damage to cells.^[^ ^24^ ^]^ To test whether γGC‐MPs worked in this process, the intracellular Fe^2+^ concentration and ROS levels were visualized using confocal microscopy (Figure 5B–D). Cells treated with γGC‐MPs were profoundly depleted of intracellular unstable Fe^2+^ and excessive ROS, implying MPs were involved in the mentioned modulatory procedure. Besides total ROS, the scavenging capabilities of γGC‐MPs against several specific species of ROS, including •OH, •O_2_^–^, and DPPH, which were key ROS involved in UC,^[^ ^25^ ^]^ were evaluated. The results affirmed the strong scavenging activity of γGC‐MPs (Figure S5, Supporting Information). Subsequently, flow cytometry results (Figure 5E,F) demonstrated that the γGC‐MPs‐containing group dramatically induced THP‐1 cells differentiation to a more M2‐like phenotype (biomarker: CD206), instead of more M1‐like ones (biomarkers: CD86 and TNF‐α), compared to free γGC treated group. As expected, the further IF staining assay corroborated these findings. Accompanied by free γGC or γGC‐MPs treatments, the expressions of TNF‐α and GPX4 were all effectively reversed compared to the model group (Figure 5G), while the γGC‐MPs group generated more potent efficacy. Furthermore, immunoblot analysis data demonstrated that γGC‐MPs treatment effectively inhibited the level of TNF‐α, the phosphorylation of PI3K and AKT2 (Figure S6, Supporting Information). Overall, due to the advantages of a large specific surface area and superior sustained release capacity, γGC‐MPs displayed more robust abilities of remodeling macrophage polarization and anti‐ferroptosis, outperforming free γGC treated alone.

### 2.5 γGC‐MPs Exhibited Excellent Therapeutic Effectiveness in the DSS‐Induced Colitis

**Figure 6:**
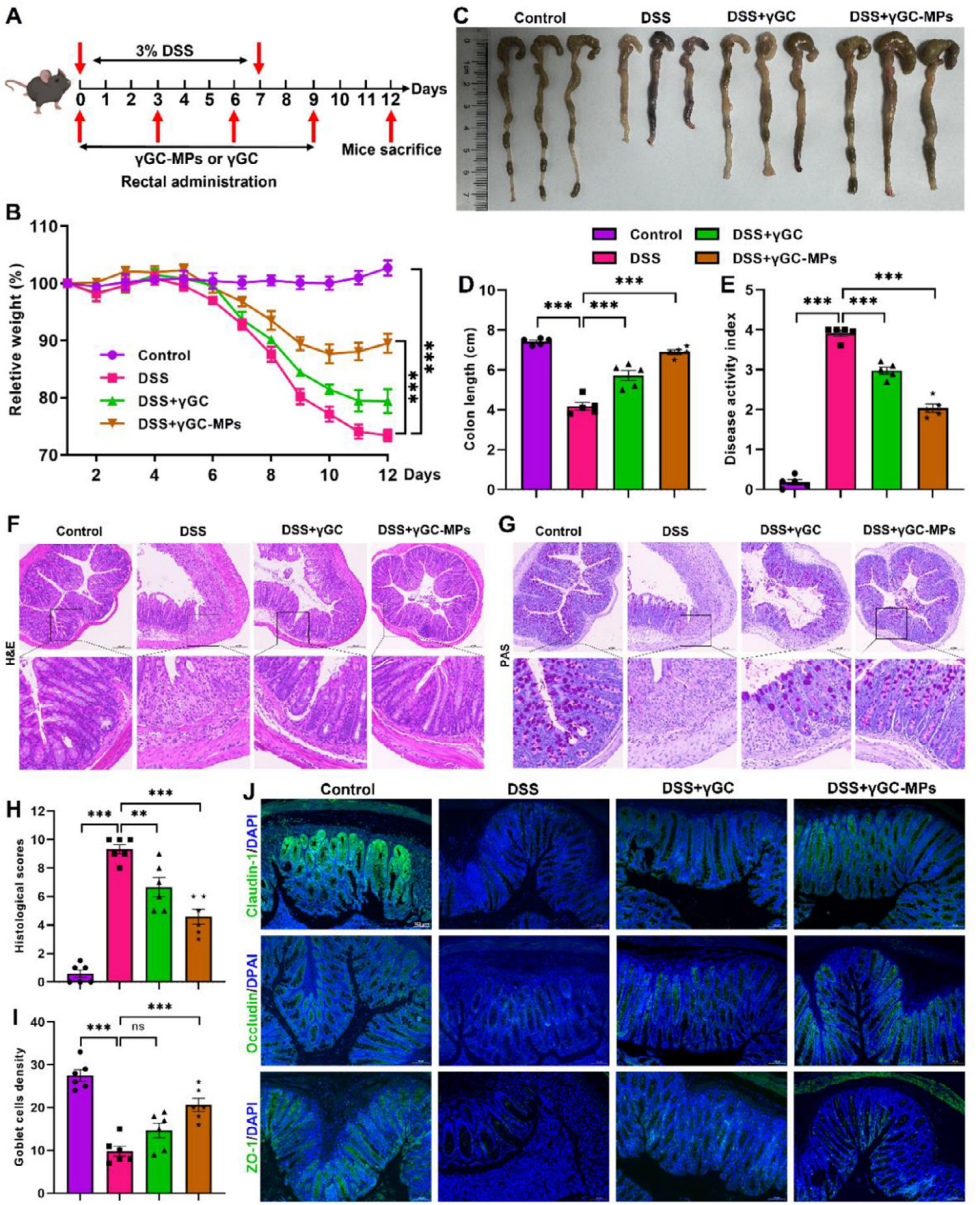
The γGC‐MPs significantly reduced DSS‐induced UC symptoms in mice. A) A schematic presentation of the drug intervention strategy. Measurements and calculations were made for relative body weight (B), macroscopic appearances (C), colon length (D), and disease activity index (E) across the various formulations. Representative sections followed by H&E (F) and PAS (G) staining were displayed (scale bar: 200 µm). Histological scores (H) and goblet cell density (I) were analyzed and calculated. J) IF images of ZO‐1, claudin‐1, and occludin in colon tissues were presented (the scale bar: 100 µm). Data are mean ± SEM. *P < 0.05, **P < 0.01, and ***P < 0.001, ns = no significant.

Effective management of UC necessitates sustaining elevated medication levels at the lesion sites, which positions adhesive drug delivery systems as optimal options for treatment.^[^ ^20^ ^]^ Encouraged by the potency of γGC‐MPs displayed in vitro, we investigated the therapeutic potential of γGC‐MPs in DSS‐induced acute colitis. Mice were administered DSS in their drinking water for 7 days, followed by 5 days of recovery with normal water. During this period, γGC or γGC‐MPs were administered rectally every three days (**Figure** **6**A). Colitis severity was evaluated by examining body weight loss, fecal characteristics, and the presence of blood in stools, with a comparison of the disease activity index (DAI) among the different groups. As anticipated, there was a reduction in body weight across all mice treated with DSS; however, a notable trend toward recovery was observed in mice receiving γGC or γGC‐MPs. Mice treated with γGC or γGC‐MPs showed significant recovery in colon length, congestion, edema, and DAI compared to DSS‐only mice (Figure 6B–E; Figure S7, Supporting Information). Note that γGC‐MPs treatment exhibited better therapeutic efficacy compared to direct intervention of γGC. Typically, the histological characteristics associated with colitis encompass the infiltration of inflammatory cells, a reduction in intestinal villi length, bending of glandular ducts, and a decrease in both goblet cells and crypts.^[^ ^1^ ^]^ H&E and PAS staining confirmed that the presence of γGC protected the colonic barrier from pathological damage, with γGC‐MPs showing superior efficacy (Figure 6F,G). A board‐certified pathologist conducted histopathological scoring of colon tissues through a validated system designed to evaluate mucosal loss, hyperplasia, inflammation, and extent. Mice treated with γGC‐MPs had the lowest histopathological scores and the highest goblet density of all treatment groups (Figure 6H,I). The integrity of the epithelial barrier is vital for proper intestinal health and physiological functioning. Key intestinal tight junction (TJ) proteins, such as ZO‐1, claudin‐1, and occludin, play an essential role in sustaining the integrity of the intestinal barrier.^[^ ^26^ ^]^ Consistent with these findings, the IF staining assay revealed that γGC‐MPs significantly increased the expressions of ZO‐1, claudin‐1, and occludin in colitis tissues compared to free γGC treatment. This suggested that γGC‐MPs effectively normalized TJ protein expression and repaired the DSS‐inflamed intestinal barrier in DSS‐induced mice.

### 2.6 γGC‐MPs Reshaped Macrophage In Vivo via Regulating PI3K/AKT Pathway and Inhibiting Ferroptosis to Rescue UC

**Figure 7:**
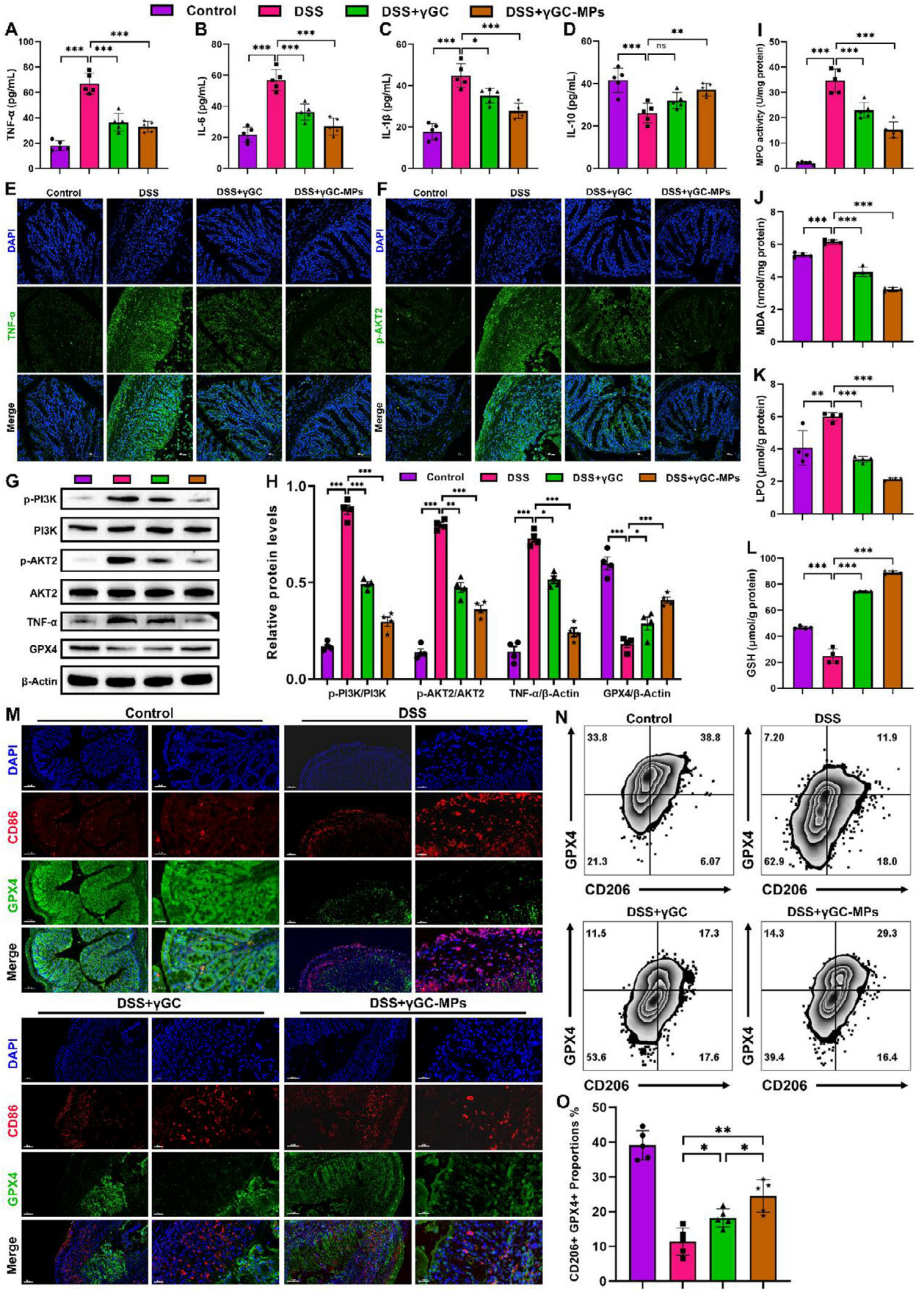
γGC‐MPs alleviated UC by inhibiting ferroptosis and modulating the PI3K/AKT signaling pathway in macrophages. A–D) The levels of TNF‐α, IL‐6, IL‐1β, and IL‐10 in colonic tissues from various groups were measured. E,F) Immunofluorescence images of TNF‐α and p‐AKT2 in colon tissues (scale bar: 50 µm). G,H) Protein expressions of p‐PI3K, PI3K, p‐AKT2, AKT2, GPX4, and TNF‐α were examined by immunoblot I–L) Measurements of MPO, MDA, LPO, and GSH levels in colonic tissues from different groups. M) Dual IF staining for CD86 and GPX4 in colonic tissues (scale bar: 30 µm). N,O) Expressions of GPX4 and CD206 in mice colon macrophages were examined by flow cytometry. Data are mean ± SEM. *P < 0.05, **P < 0.01, and ***P < 0.001, ns = no significant.

Reshaping macrophage polarization is beneficial for restoring the colon barrier and reducing the inflammatory response in the treatment of UC.^[^ ^10^ ^]^ Consequently, relevant studies were conducted to explore whether γGC‐MPs could enhance anti‐inflammatory effectiveness and maintain homeostasis in the colon by modulating macrophage polarization. Both γGC‐MPs and free γGC treatments notably decreased the secretion of pro‐inflammatory cytokines (IL‐6, IL‐1β, and TNF‐α) while simultaneously increased the expression of anti‐inflammatory cytokine IL‐10, compared to the DSS group (**Figure** **7**A–D). Consistently, IF staining and immunoblot analysis showed that γGC‐MPs markedly inhibited the phosphorylation of pro‐inflammatory factors, PI3K, and AKT2, outperforming free γGC treated alone (Figure 7E–H). Moreover, γGC‐MPs significantly upregulated GPX4 expression compared to the DSS group (Figure 7G,M). GPX4, serving a gatekeeper of ferroptosis, plays a crucial role in limiting lipid peroxidation.^[^ ^27^ ^]^ The levels of relevant markers, myeloperoxidase (MPO), malondialdehyde (MDA), and lipid peroxidation (LPO) in colon tissue were significantly reduced after γGC‐MPs or free γGC treatment compared to the UC group (Figure 7I–K). The addition of γGC‐MPs or free γGC significantly increased GSH production in colon of colitis mice (Figure 7L).

To assess the effect of γGC‐MPs referring to ferroptosis susceptibility in M1/M2 subsets in vivo, dual IF staining and flow cytometry analysis were performed to correlate GPX4 expression with macrophage subtypes in the colon post‐treatment (Figure 7G–M). The results indicated that γGC‐MPs treatment significantly reduced the proportion of M1 macrophages (CD86) while enhancing the proportion of M2 macrophages (CD206) in comparison to the DSS group, suggesting that γGC‐MPs facilitated the shift in macrophage polarization from the M1 to M2 phenotype (Figure 7M–O; Figure S8, Supporting Information). Meanwhile, the expression of GPX4 was found to be positively associated with the M2 phenotype, while showing a negative correlation with the M1 phenotype, highlighting that anti‐ferroptosis treatment for macrophage reprogramming could offer new therapeutic targets for UC treatment.

Finally, the biosafety of γGC‐MPs was validated via H&E staining analysis (Figure S9, Supporting Information). The results showed that the main organs (heart, liver, kidneys, lungs, and spleen) did not show notable structural variations or pathological changes following the treatment (Figure S9A, Supporting Information). Furthermore, the assessment of serum physicochemical parameters (alanine aminotransferase (ALT), aspartate aminotransferase (AST), blood urea nitrogen (BUN), and creatinine (Cr)) indicated no significant discrepancies (Figure S9B–E, Supporting Information), referring to the excellent biosafety properties of γGC‐MPs. Further, we administered γGC‐MPs to the mice for long‐term toxicity studies, and no abnormality was found after 14 and 28 days of examination of t H&E staining analysis and serum physicochemical parameters (Figure S9F–J, Supporting Information).

### 2.7 γGC‐MPs Optimized Gut Microbiome to Assist UC Treatment

**Figure 8:**
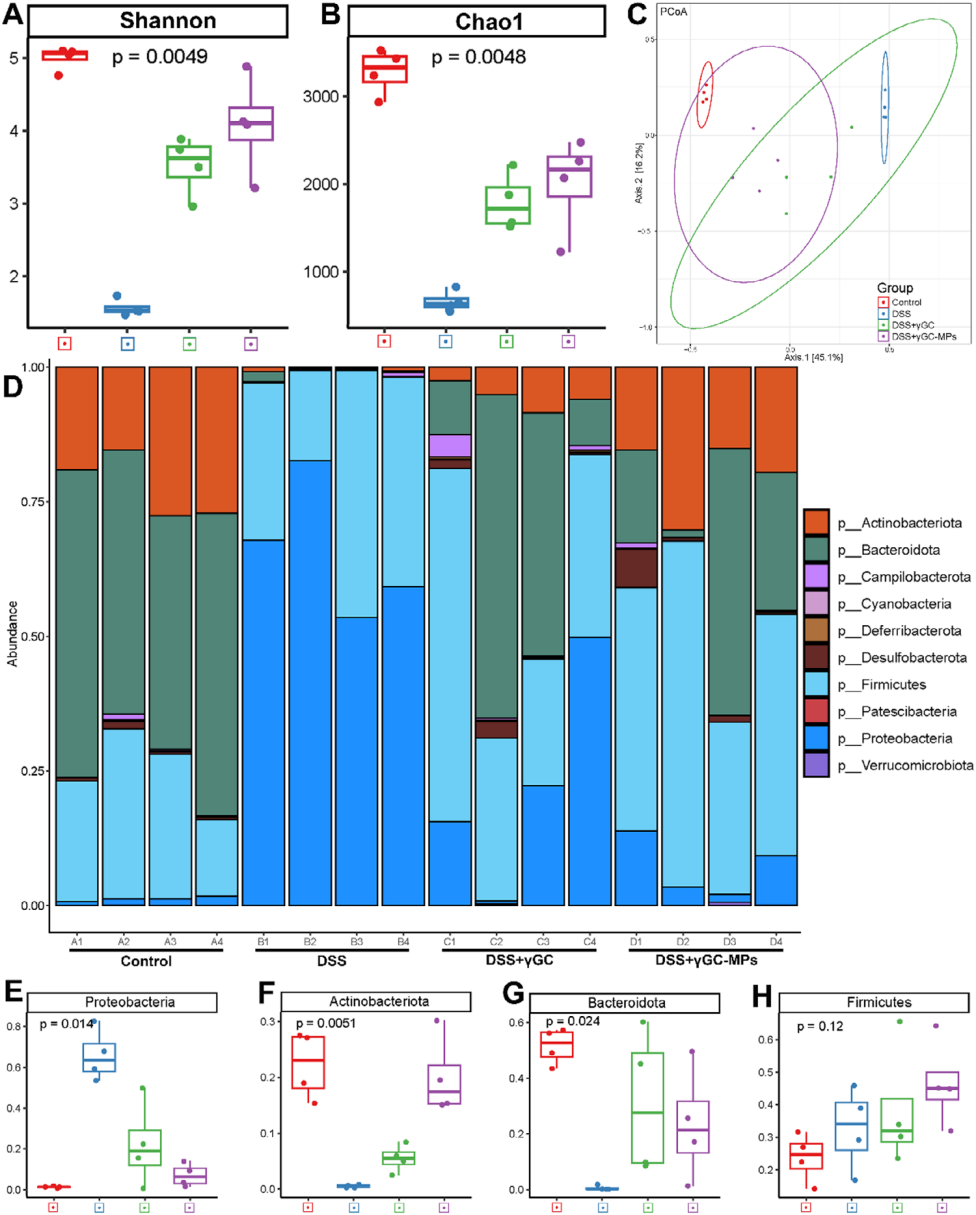
The γGC‐MPs restored the balance of intestinal flora in DSS‐induced UC mice. A,B) The α diversity indices, Shannon and Chao1 were analyzed. C) The gut microbiome β diversity was illustrated by PCoA. D) Histogram of phylum abundance among groups. E–H) The relative abundance of specific taxa at the phylum level was assessed following various treatments. Statistical analysis of the gut microbiome was calculated by the Kruskal–Wallis test (*P < 0.05).

It has been reported that the pathogenesis of UC is tightly linked to the imbalance of gut commensal microbiota. To investigate the regulatory impacts of γGC‐MPs on the gut microbiome, fecal samples underwent 16S rRNA gene amplicon sequencing for a thorough analysis of microbiome composition. Analysis of α diversity revealed there were notable variations in the diversity and abundance of intestinal microorganisms in DSS‐induced colitis mice, as assessed by Shannon and Chao1, in comparison to the other three groups (**Figure** **8**A,B). The principal co‐ordinates analysis (PCoA) indicated a clear separation between colitis mice and healthy mice. However, the intestinal microbiota profile of γGC‐MPs‐treated mice closely resembled that of normal mice, which suggested a significant alteration in the colitis microbiota after treatment (Figure 8C). A Venn diagram was performed to distinguish the data regarding both the shared and distinct taxa of the intestinal microbiota across the four groups (Figure S10, Supporting Information). Following this, an in‐depth analysis of the gut microbiome was conducted.

At the phylum level, DSS treatment notably raised the relative abundance of Proteobacteria,^[^ ^25^, ^28^ ^]^ which serves as a microbial indicator of dysbiosis in patients with UC. Actinobacteriota, Bacteroidota, and Firmicutes are considered beneficial gut bacteria.^[^ ^29^, ^30^ ^]^ There were significant changes in both bacterial diversity and community composition of mice gut after various treatments. In particular, free γGC or γGC‐MPs treatments decreased the abundance of Proteobacteria in colitis mice and effectively enhanced the relative abundance of Actinobacteriota and Bacteroidota (Figure 8D–H; Figure S11, Supporting Information). γGC‐MPs treatment also prominently increased the relative levels of Firmicutes but showed no significant influence on the abundance of Firmicutes.

To further clarify the differences noted in microbiome composition, we conducted a linear discriminant analysis effect size (LEfSe) evaluation (Figures S12,S13, Supporting Information). Consistent with the findings mentioned earlier, there was a significant reduction in the abundance of Bacteroidota alongside a marked increase in Proteobacteria and Enterobacteriaceae associated with UC, specifically *Escherichia shigella* and *Escherichia coli*, in colitis mice (LDA score > 3, *P* < 0.05). However, after applying γGC‐MPs, there was a decrease in the number and species of these pathogens, while the level of beneficial genus Faecalibaculum saw a substantial increase compared to other groups, demonstrating the capacity of γGC‐MPs to effectively address dysbiosis. All these changes proved that γGC‐MPs efficaciously modulated and optimized the intestinal microbiota composition in UC mice. A deeper exploration is necessary to further understand the exact mechanisms that govern the relationship between γGC‐MPs and the gut microbiome.

## 3 Discussion

UC represents a persistent inflammatory condition affecting the gastrointestinal system, substantially diminishing the quality of life for affected individuals and presenting a growing challenge to public health worldwide.^[^ ^31^ ^]^ The dysregulated immune response and intestinal cell metabolism are typical pathological features of colitis. Macrophages, highly plastic immune cells, exhibit remarkable phenotypic and metabolic flexibility, adapting to their specific spatial and temporal pathophysiological environment.^[^ ^10^, ^32^ ^]^ Macrophages, which play a crucial role in the innate immune system, are essential for the onset of UC^[^ ^33^ ^]^ and drive focal inflammation as well as UC progression via polarizing to the more M1‐like phenotype. Abnormal macrophage survival in the colon contributes to the persistent progression of colitis and has been utilized for targeted therapies.^[^ ^34^ ^]^ For instance, Janus nanomotors coated with M2 macrophage membranes made of sodium alginate microspheres have effectively targeted macrophages in UC, depleted reactive oxygen species, and shown promising efficacy in colitis mice.^[^ ^30^ ^]^ Additionally, ferroptosis is observed in immune cells within pathological settings of inflammation to varying extents. Given that, the present study attempted to explore the underlying junction points between the occurrence of ferroptosis and macrophage polarization during UC progression and to target these connection points for UC alleviation.

Altered gut metabolites in UC patients were identified at first in our study. γGC, a direct precursor of GSH, was noted to be significantly downregulated in the UC colon (Figure 2). Meanwhile, our data revealed severe M1 macrophage infiltration in colon tissues of patients with UC and a positive correlation between ferroptosis and M1 phenotype in gut macrophages (Figure 2). Meanwhile, as data revealed severe M1 macrophage infiltration in colon tissues of UC patients and there was a positive correlation between ferroptosis and M1 phenotype in gut macrophages, we suspected that it was γGC deletion‐mediated ferroptosis susceptibility in the colon disturbed the normal homeostasis of macrophages in human UC. Intracellular GSH deficiency is considered a characteristic of ferroptosis. As the precursor to GSH, exogenous γGC enters cells and is enzymatically converted by GSS, thereby enhancing intracellular GSH concentrations to combat ferroptosis. Importantly, γGC addition does not trigger allosteric feedback inhibition of GSS activity.^[^ ^35^ ^]^ Studies have shown that the administration of γGC safeguarded mice from toxicity induced by CLP or LPS, with its anti‐inflammatory mechanism involving elevated intracellular GSH levels and reduced ROS accumulation.^[^ ^17^ ^]^ Consistently, we demonstrated that γGC supplementation markedly inhibited ferroptosis and modulated the PI3K/AKT signaling pathway, thereby suppressing the polarization of pro‐inflammatory M1 macrophage (Figure 3). Consequently, γGC was utilized to replicate the natural defense systems against oxidative stress and ferroptosis, serving as a substitute for traditional anti‐inflammatory medications.

The management of UC requires elevated levels of medication at the lesion sites, which renders adhesive drug carriers particularly appropriate. Nonetheless, the in vivo administration of free γGC to induce macrophage polarization in specific tissues encounters considerable challenges, including poor pharmacokinetics, limited accumulation in the lesion regions, and nonspecific target capacity. Moreover, γGC is highly susceptible to deactivation in the harsh gastrointestinal environment, impairing its ability to regulate complicated inflammatory responses in UC. Emerging MPs‐based delivery systems aim to address these challenges by enhancing drug release and accumulation at local lesions.^[^ ^25^, ^36^ ^]^ For instance, magnetic biological robots, developed by evaporating a layer of ferromagnetic metal onto MPs of defatted sunflower pollens, utilize electrostatic forces to maximize their accumulation in tumor sites for enhanced efficacy in combating malignancies.^[^ ^21^ ^]^ Another nanomedicine designed for oral administration, which incorporated TNF‐α–small interfering RNA alongside gallic acid‐mediated graphene quantum dots, then encapsulated within bovine serum albumin NPs, layered with chitosan and tannic acid. Its efficacy in modulating immune responses and altering gut microbiota in cases of acute colitis has been demonstrated.^[^ ^37^ ^]^ Given that UC often impacts the rectum and terminal section of the colon, oral administration may result in premature release and off‐target effects.^[^ ^38^ ^]^ Enema administration is thereby chosen as it is one of the most commonly used approaches for UC treatment in clinics.^[^ ^20^ ^]^ Inspired by the geometrical morphology of sunflower pollens, an adhesive carrier featuring a unique claw‐engaged structure was constructed for delivering exogenous γGC, accomplishing the enema‐based treatment of UC (Figure 4). The resulting γGC‐MPs exhibited favorable physicochemical properties and excellent biocompatibility. The increased specific surface area and claw‐engaged adhesive capacity of MPs extended the retention time of γGC. γGC‐MPs group significantly alleviated severe UC symptoms in mice with DSS‐induced colitis compared to the free γGC treated group, as evidenced by decreased intestinal inflammation and suppressed ferroptosis, macrophage reprogramming, enhanced ROS scavenging, and the restoration of both the intestinal barrier and the microbiota.

Current clinical treatment strategies for UC, including anti‐inflammatory agents, immunosuppressants, and biologics, remain limited by systemic toxicity, incomplete mucosal healing, and high relapse rates.^[^ ^2^, ^39^ ^]^ While biologics targeting TNF‐α or integrins have improved clinical outcomes, their efficacy is often compromised by immunogenicity and secondary non‐response,^[^ ^40^, ^41^ ^]^ whereas JAK inhibitors raise safety concerns over thromboembolic risks.^[^ ^39^, ^42^ ^]^ Moreover, conventional therapies predominantly focus on inflammatory suppression without addressing redox imbalance or microbiota dysbiosis—key drivers of UC progression. In contrast, our γGC‐MPs platform introduces a multifaceted approach by synergistically targeting macrophage ferroptosis, polarization, and gut microbiota remodeling. Unlike synthetic nanocarriers, the natural pollen chassis ensures biocompatibility and scalability, circumventing allergenic risks.^[^ ^43^ ^]^ It should be acknowledged that there still exist several restrictions regarding the potential clinical translation of this study. Given the persistent and recurring characteristics of UC, our study only evaluated the short‐term efficacy of γGC‐MPs within an acute UC model, which may not fully represent the long‐term treatment needs for chronic UC. Additionally, the absence of large animal models limits the study's ability to address the complex pathology of human UC, as mouse models have inherent limitations. Furthermore, the cost and quantification of γGC could pose challenges in good manufacturing practice settings due to stringent production requirements.

## 4 Conclusion

In summary, this study confirmed that γGC deletion in UC lesions served as the key driver for exacerbating macrophage reprogramming by promoting macrophages ferroptosis to induce inflammation. Replenishing exogenous γGC significantly decreased the ratio of M1 macrophage polarization by inhibiting ferroptosis, modulating the PI3K/AKT pathway and TNF signaling pathway. To maximize the accumulation of γGC in the intestinal lesion area, an advanced MPs‐based delivery system based on sunflower pollens with a porous and spiky structure was constructed to load γGC. The utilization of γGC‐MPs indeed enhanced the possibility to contact lesion sites, as well as improved γGC focal release and its absorption by tissues. In a DSS‐induced colitis model mice, γGC‐MPs administration markedly alleviated severe UC symptoms and optimized gut microbiome. Overall, this multifaceted strategy highlights the substantial potential of γGC‐MPs for UC treatment, setting the stage for further research in exploring mechanisms involving UC progression and developing novel therapeutics to target clinical translation.

## 5 Experimental Section

### Clinical Specimen Collection

The colon samples were taken from patients who underwent surgical resection. From January 2023 to January 2024, 8 patients with pathologically diagnosed UC (5 frozen samples and 4 paraffin‐embedded samples) and 8 volunteers without intestinal disease (5 frozen samples and 4 paraffin‐embedded samples) were enrolled in this study (Table S1, Supporting Information). The Institutional Review Board of the First Affiliated Hospital of Nanjing Medical University conducted a review and approved the research protocol and its ethical considerations (2024‐SR‐489).

### Metabolomic Analysis

Human intestinal mucosa samples were collected from volunteers without intestinal disease (*n* = 4), UC patients (*n* = 4), in accordance with predefined inclusion and exclusion criteria, for untargeted metabolomics analysis (Biozeron, Shanghai, China). The samples were subjected to grinding with liquid nitrogen, and the resultant homogenates were then resuspended in prechilled 80% methanol that contained 0.1% formic acid, ensuring thorough vortex mixing. After 5 min of incubation on ice, centrifugation was performed at 4 °C for another 5 min. A portion of the resulting supernatant was diluted with LC‐MS grade water to reach a final methanol concentration of 53%. The samples were subsequently transferred to new Eppendorf tubes and centrifuged at 15 000 g for 10 min at 4 °C. The supernatant was then analyzed by injecting it into the LC‐MS/MS system. The raw data files produced by UHPLC‐MS/MS were subjected to processing using Compound Discoverer 3.1 (CD3.1, Thermo Fisher) for tasks such as peak alignment, peak picking, and metabolite quantification. The analysis of metabolite profiles and their differences was carried out through partial least squares discriminant analysis (PLS‐DA) between the specified groups (UC versus Control). |LDA score| > 4 and *P* < 0.05 were established as criteria for identifying significantly differential metabolites.

### RNA Sequencing (RNA‐Seq)

Human intestinal mucosa samples were obtained from volunteers, comprising four individuals without any intestinal diseases and four patients diagnosed with UC. The selection of these participants adhered strictly to predefined inclusion and exclusion criteria specifically established to conduct transcriptome sequencing. This process was facilitated by LC‐Bio Technologies located in Hangzhou, China. RNA was isolated from the endoscopic samples utilizing TRIzol (Invitrogen, CA, USA) following the manufacturer's instructions. The preparation of cDNA libraries was carried out with the TruSeq RNA Library Prep Kit (Illumina, USA), followed by sequencing on a HiSeq platform (Illumina). Gene expression data was obtained using the featureCounts module of the subread package (v2.0.3). The DESeq2 package was employed to perform differential expression analysis, identifying differentially expressed genes based on criteria of |log_2_FC| ≥ 1 and adjusted *P* < 0.05.

### Isolation of Colon Macrophages in Mice

Macrophages were extracted from murine colons following established protocols.^[^ ^44^ ^]^ After the washing process, the colon was immersed in 20 mL of collagenase digestion buffer, which contained 100 U mL^−1^ of collagenase E derived from Clostridium histolyticum mixed with 40 mL of colon buffer, and incubated at 37 °C for 60 min. Following this, the supernatant was filtered through a 40 µm cell strainer. To halt the enzyme activity, chilled colon buffer (500 mL RPMI, 10 mm HEPES, 10% FBS) was introduced. The leftover tissue fragments were disaggregated, and the resulting suspension was passed through another 40 µm cell strainer. The resulting mixture was centrifuged at 800 g for 5 min at 4 °C, and the pellet was collected. For the isolation of mononuclear cells, density gradient centrifugation was performed utilizing 44% and 66% silica‐based separation media, running at 859 g for 25 min without deceleration. The mononuclear cells, appearing as a white band 1—2 mm thick, were then gathered for further experiments.

### Cell Culture, Fluorescence Imaging, and siRNA Transfection

THP‐1 cells were cultured in RPMI 1640 medium (11 875 093, ThermoFisher), enriched with 10% fetal bovine serum (FBS, JYK‐FBS‐303) and 1% penicillin‐streptomycin (15‐140‐122, Gibco). The incubation occurred in a humid environment at 37 °C with 5% CO_2_. To promote differentiation of THP‐1 cells into M0 macrophages, phorbol 12‐myristate 13‐acetate (PMA, 16561‐29‐8, Merck) was administered at a concentration of 100 ng mL^−1^. M1 macrophage polarization was induced by stimulating PMA‐differentiated cells with lipopolysaccharide (LPS, L2387, Merck) and interferon‐gamma (IFN‐γ, IF002, Merck) for 18–24 h.

For the purpose of fluorescence imaging, the cells were plated on coverslips in single‐well dishes at a density of 1 × 10^5^ cells mL^−1^. After different treatments, the fluorescence signals of fixed cells were visualized by microscopy.

In a separate experiment, the negative control and GSS siRNA (HY‐RS05871, MCE, USA) were transfected into cells following the provided manufacturer's guidelines and subsequently collected cells for further analysis.

### Preparation of Pollen γGC‐Microparticles (γGC‐MPs)

Pollen grains from natural sunflowers (*Helianthus annuus*) were gathered. γGC, dopamine (DA) hydrochloride, chitosan quaternary ammonium salt, calcium chloride, and sodium alginate were purchased from Sigma Aldrich Co. LTD. The Fe3O4 magnetic nanoparticles (NPs) were bought from Nanjing Nanoeast Biotech Co. LTD.

γGC‐MPs were prepared as follows:^[^ ^1^ ^]^ The natural pollens were first washed with excess ethanol solution and collected by centrifugation. The above steps were repeated five times to achieve favorable dispersion of pollen MPs.^[^ ^2^ ^]^ The DA solution was then added to the raw pollen/water dispersion with excess PDA removed by four times of water rinse after stirring at room temperature overnight.^[^ ^3^ ^]^ Fe_3_O_4_ magnetic NPs dispersion (100 µL) was added to a 5 mL mixture system and shaken for 4 h to allow the NPs to fully adsorb onto the pollen surface.^[^ ^4^ ^]^ The RP@Fe MPs were then calcined at 600 °C with sustained nitrogen supply for 12 h to obtain shrunken porous spike‐like CRP@Fe templates.^[^ ^5^ ^]^ CRP@Fe templates (10 mg) were immersed in chitosan quaternary ammonium salt solution (5 mg mL^−1^, 5 mL) with an appropriate amount of γGC and stirred for 4 h at room temperature. After water rinsing to remove the excess chitosan, the above particles were immersed into the negatively charged alginate solution (5 mg ml^−1^, 5 mL) for 4 h. Then, water rinsing was applied to remove the extra alginate.^[^ ^6^ ^]^ Finally, γGC‐MPs were obtained after the rinsing step with water and then stored at 4 °C for further experiments. FITC (fluorescein isothiocyanate)‐labeled MPs were prepared by adding CRP@Fe@γGC particles into the FITC solution. The in vitro release study was conducted using artificial colonic fluid containing phosphate, ammonia, and so on, which was purchased from Shanghai Lianmai Biological Engineering Co. LTD. The pH of the simulated intestinal fluid was adjusted to the range of UC patients’ diseased colon, which is ≈2.7 to 5.5.

### Characterization of γGC‐MPs

The scanning electron microscope (SEM, Hitachi S3000 N) with a gold sputter coating was used to characterize the microstructures of the MPs and pollens. Optical images were captured using a stereomicroscope (NOVEL NTB‐3A, Ningbo Yongxin Optics Co., Ltd.) and documented with a CCD (Media Cybernetics Evolution MP 5.0 RTV). The diameter of the MPs was assessed using AOS Imaging Studio V3.4.2 software. X‐ray photoelectron spectroscopy (XPS) was performed using the E03‐001 scanning focusing multifunctional platform (PHI 5000 VersaProbe III). X‐Ray Diffraction (XRD) X‐ray diffraction measurements were investigated through a Rigaku UItima IV X‐ray diffractometer (Tokyo, Japan). Fourier transform infrared (FT‐IR) spectra over a range of 500 to 4000 cm^−1^ were recorded by FT‐IR spectroscopy (Nicolet iS50, Thermo Fisher Scientific, USA). Additionally, variations in zeta potential were measured utilizing a dynamic light scattering system (Malvern Panalytical Zetasizer Nano ZS90, UK).

### Cell Viability Analysis

The assay for cell counting kit‐8 (CCK‐8, MCE, USA) was conducted following the guidelines provided by the manufacturer. Cells were plated in 96‐well plates at a density of 5 × 10^4^ mL^−1^. Following differentiation, cells received treatment with 100 µL of fresh medium that included various concentrations of γGC or γGC‐MPs for a duration of 24 h. After an incubation period of 2 h at 37 °C with CCK‐8 (10 µL), the absorbance was recorded at 450 nm utilizing a microplate reader (Tecan, Switzerland).

### Flow Cytometry

Briefly, the cell suspension was treated with blocking buffer at 4 °C for 30 min. For cell surface proteins, a 30‐minute incubation with primary antibody was sufficient for detection. For intracellular proteins, cells needed to be fixed and permeabilized followed by primary antibody incubation. The details of antibodies used for flow cytometry are provided in Table S2 (Supporting Information). CytoFLEX Flow Cytometers (Beckman Coulter) were used to detect the fluorescence intensity of different proteins. Data analysis was performed by FlowJo software (v10.8, BD Biosciences).

### Determination of Intracellular Fe^2+^ Levels

THP‐1 cells activated by PMA were placed into a 48‐well plate. Following the guidelines provided by the manufacturer, the cells underwent treatment with LPS and IFN‐γ for a duration of 24 h. Subsequently, they were rinsed three times using HBSS and treated with either γGC or γGC‐MPs (200 µm) for another 24 h. After this incubation period, the cells were stained with 1 µm FerroOrange (Dojindo, Kumamoto, Japan) in HBSS for 30 min at 37 °C, and then immediately observed using a fluorescence microscope (OLYMPUS).

### Assessment of Intracellular ROS‐Scavenging Capability

THP‐1 cells differentiated using PMA were treated with LPS and IFN‐γ, then incubated for 24 h with either γGC or γGC‐MPs at a concentration of 200 µm. After the media was discarded, each well received 500 µL of a 10 µm DCFH‐DA solution, which was allowed to incubate for 30 min. Subsequent to three gentle rinses, the levels of intracellular ROS were evaluated by examining DCF green fluorescence signals through a fluorescence microscope (OLYMPUS).

### Establishment of DSS‐Induced Colitis Model and Treatment

The protocols for animal experimentation adhered to the guidelines established by the National Institutes of Health for the care and utilization of laboratory animals and received approval from the Medical Ethics Committee at Nanjing Normal University (IACUC‐2024240). Seven‐week‐old female C57BL/6 mice were sourced from Gempharmatech (Nanjing, China). Following one week of cohabitation, the mice were randomly assigned to various groups to ensure synchronization of their gut microbiomes.

The subjects were distributed into four distinct groups (*n *= 5): The healthy control group (G1), the DSS group (G2), the DSS + γGC group (G3), and the DSS + γGC‐MPs group (G4). The control group was provided with sterile drinking water, while the remaining groups received a 3% (w/v) solution of dextran sulfate sodium (DSS) (MP Biomedicals, Solon, OH, USA) in their drinking water for a period of seven days to induce acute colitis. Throughout the experiment, mice in groups G3 and G4 were administered γGC (500 mg k^−1 ^g) or γGC‐MPs (500 mg k^−1 ^g) rectally every three days. Daily monitoring was conducted to observe changes in body weight, stool consistency, and instances of fecal bleeding. The DAI was computed to evaluate the severity of symptoms, aggregating scores for weight loss (0–4), stool consistency (0–4), and fecal bleeding (0–4).^[^ ^45^ ^]^ On day 12 after the treatment, all mice were euthanized, and samples of serum, heart, liver, spleen, lungs, kidneys, and colon were meticulously collected for further analysis.

To further verify that γGC‐MPs could promote the intestinal retention of γGC, FITC‐labeled MPs were employed. Mice were euthanized and their abdomen was dissected 12‐, 24‐, and 36‐hours post‐administration, respectively. Images were obtained in the supine position using an IVIS Lumina III in vivo imaging system (Excitation, 480 nm; Emission, 520 nm).

### H&E and PAS Staining

Colonic specimens were preserved in 4% formalin for a duration of 48 h. Following this fixation process, the samples were sequentially submerged in ethanol solutions of 70%, 80%, and 90% for 30 min each, which was then succeeded by treatments in 95% and 100% ethanol, each applied twice for 20 min. Once dehydrated, the specimens underwent infiltration with xylene, were embedded in paraffin, and then sectioned to a thickness of 5 µm. Staining was conducted using Hematoxylin–Eosin (H&E) and Periodic Acid‐Schiff (PAS), after which analysis was carried out using a light microscope (Olympus, Tokyo, Japan). The histological scoring for colitis was calculated by summing the severity of inflammation (0–3), crypt damage (0–3), ulceration (0–3), and edema (0–3), as outlined in previous research.^[^ ^45^ ^]^

### Immunofluorescence (IF) Staining

After deparaffinization and gradient hydration, sections were processed for antigen retrieval and blocking of endogenous peroxidase activity. A blocking buffer (10% FBS) was then applied to block non‐specific staining for 1 h. Afterward, primary antibodies were incubated overnight at a temperature of 4 °C. Subsequently, all slides were treated with fluorescence‐conjugated secondary antibodies for a duration of 30 min, and this was followed by a 5‐minute DAPI staining process. The resulting stained slides were examined using an Olympus BX53 system.

### Immunocytochemical (ICC) Staining

Cells were treated with 4% formaldehyde for a duration of 30 min for fixation. Following this process, Triton X‐100 (9036‐19‐5, Merck) was used to permeabilize the cells, and they were then incubated with 1% BSA to prevent non‐specific binding. The primary antibodies were applied to the cells and incubated overnight at 4 °C, after which the corresponding secondary antibodies were applied and incubated at room temperature for 1 h. To counterstain the cell nuclei, DAPI was utilized, and imaging was performed using fluorescence microscopy (Axio Scope). The antibodies used are listed in Table S3 (Supporting Information).

### Western Blot

Colonic tissues and cells were lysed using RIPA lysis buffer (Beyotime) to obtain total protein for Western blot (WB) analysis. SDS‐polyacrylamide gel electrophoresis was performed according to previously established methods.^[^ ^46^ ^]^ PVDF membranes were incubated at room temperature for 1 to 2 h with a blocking solution of 10% nonfat milk and then underwent overnight incubation at 4 °C with primary antibodies diluted (1:1000) in QuickBlock Primary Antibody Dilution Buffer (Beyotime). Following this, secondary antibodies (1:40 000) were applied for one hour at room temperature. After three rinses with TBST, the bands were visualized with Western chemiluminescent HRP substrate (Millipore, Germany). β‐actin served as the internal control for normalizing protein levels. Chemiluminescence signals were captured with a Tanon 5200‐Multi Chemiluminescent Imaging System (Tanon, China). Densitometric analysis and β‐actin normalization were conducted using ImageJ. Band density was calculated and statistically analyzed using ImageJ software. The antibodies used are listed in Table S4 (Supporting Information).

### Quantitative Real‐Time Polymerase Chain Reaction (RT‐qPCR)

Total RNA was isolated from all samples employing TRI Reagent (Takara). The synthesis of cDNA was carried out using the High‐Capacity cDNA Reverse Transcription Kit (Thermo Fisher Scientific). SYBR Green qPCR Kits (Vazyme Biotech, Nanjing, China) were utilized to perform quantitative PCR (qPCR). The relative expression levels of target genes were normalized with β‐actin serving as the reference gene. Details of the primers are available in Table S5 (Supporting Information).

### Measurement of LPO, MPO, MDA, and GSH Levels

Colon or cell samples were swiftly collected and homogenized at a temperature of 4 °C. The protein concentrations were measured quantitatively with a BCA protein assay kit (Pierce Biotechnology, Illinois, USA). The levels of lipoperoxide (LPO), myeloperoxidase (MPO), malondialdehyde (MDA), total glutathione (GSH), and oxidized GSH were assessed using specific assay kits for LPO, MPO, and MDA (Nanjing Jiancheng Bioengineering Institute) as well as a GSH assay kit (BioVision), adhering to the manufacturer's instructions.

### Radical Scavenging Assay

The scavenging activity of the •OH radical for γGC‐MPs was evaluated using the ABTS method. In summary, a mixture was prepared by combining 920 µL of H_2_O, different concentrations of γGC‐MPs (50, 100, and 200 µm), 20 µL of FeSO_4_·7H_2_O (18 mm), and 10 µL of H_2_O_2_ (200 µm). Following a 10‐minute ultrasonic treatment, the supernatant was collected through centrifugation and subsequently incubated with 50 µL of ABTS (10 µm) for a duration of 5 min. The antioxidant activity was measured by recording the UV absorbance at 800 nm.

The •O_2_^–^ radical scavenging ability of γGC‐MPs was assessed through the inhibition of NBT photoreduction. Specifically, a mixture containing 20 µm riboflavin, multiple concentrations of γGC‐MPs (50, 100, and 200 µm), 12.5 mm methionine, and 75 µm NBT was prepared in PBS. After exposing this mixture to UV light for 1 h, the antioxidant activity was quantified by measuring UV absorbance at 560 nm.

DPPH was dissolved in 100 mm 95% ethanol to prepare a stock solution. Subsequently, different concentrations of γGC‐MPs (50, 100, and 200 µm) were incubated with DPPH in the dark for 20 min. The antioxidant activity was then evaluated by measuring the UV absorbance at 515 nm.

### ELISA Analysis

Samples of the colon were obtained, promptly homogenized at a temperature of 4 °C, and the resultant supernatant was collected. Cytokine levels (TNF‐α, IL‐1β, IL‐6, and IL‐10) in these samples were assessed employing targeted mouse ELISA kits (Nanjing Kirton Biotech Co. Ltd., Nanjing, China).

### Evaluation of Biosafety In Vivo

Serum levels of aspartate transaminase (AST), alanine transaminase (ALT), blood urea nitrogen (BUN), and creatinine (Cr) were measured following the procedures outlined by the manufacturer (Jianglai Biological). H&E staining of key organs including the heart, liver, kidney, lung, and spleen, were performed to evaluate histological responses.

### Gut Microbiota Analysis

Fecal samples from mice were gathered for the extraction of total genomic DNA utilizing the E.Z.N.A. Genomic DNA Isolation Kits (Omega Bio‐Tek Inc., USA). The amplification of the V3‐4 hypervariable region of the bacterial 16S rRNA gene was performed using the universal primers 338F and 806R. Deep sequencing was carried out on the Illumina Miseq/Nextseq 2000/Novaseq 6000 platforms (Illumina, Inc., USA) at Beijing Allwegene Technology Co., Ltd. The composition of the microbiota was analyzed according to standard protocols.

### Statistical Analysis

Data are expressed as mean ± SEM and were analyzed through GraphPad Prism software. Statistical significance was set at *P* < 0.05 (*P < 0.05, **P < 0.01, and ***P < 0.001). A *t‐*test was used to evaluate differences between the two groups, whereas one‐way ANOVA, followed by Tukey's post hoc test, was employed for comparisons among multiple groups.

## Conflict of Interest

The authors declare no conflict of interest.

## Author Contributions

R.W. and J.Z. contributed equally to this work. Conceptualization was done by R.W., J.Z., and X.C. Methodology was done by R.W., J.L., J.Z., and M.W. Investigation was done by R.W., Y.W., X.C., and D.Z. Visualization was done by R.W., Y.W., J.Z., and Y.W. Supervision was done by R.W., X.C., J.Z. Original draft was written by R.W., Y.W., and J.Z. Review writing and editing was done by R.W., J.Z., and J.Z. Revision was done by R.W., J.Z., and J.Z.

## Supporting information



Supporting Information

## Data Availability

The data that support the findings of this study are available on request from the corresponding author. The data are not publicly available due to privacy or ethical restrictions.

## References

[1] C. Le Berre , S. Honap , L. Peyrin‐Biroulet , Lancet 2023, 402, 571. 37573077 10.1016/S0140-6736(23)00966-2

[2] B. Gros , G. G. Kaplan , JAMA, J. Am. Med. Assoc. 2023, 330, 951. 10.1001/jama.2023.15389 37698559

[3] C. S. Smillie , M. Biton , J. Ordovas‐Montanes , K. M. Sullivan , G. Burgin , D. B. Graham , R. H. Herbst , N. Rogel , M. Slyper , J. Waldman , M. Sud , E. Andrews , G. Velonias , A. L. Haber , K. Jagadeesh , S. Vickovic , J. Yao , C. Stevens , D. Dionne , L. T. Nguyen , A. C. Villani , M. Hofree , E. A. Creasey , H. Huang , O. Rozenblatt‐Rosen , J. J. Garber , H. Khalili , A. N. Desch , M. J. Daly , A. N. Ananthakrishnan , et al., Cell 2019, 178, 714. 31348891 10.1016/j.cell.2019.06.029 PMC6662628

[4] J. Lloyd‐Price , C. Arze , A. N. Ananthakrishnan , M. Schirmer , J. Avila‐Pacheco , T. W. Poon , E. Andrews , N. J. Ajami , K. S. Bonham , C. J. Brislawn , D. Casero , H. Courtney , A. Gonzalez , T. G. Graeber , A. B. Hall , K. Lake , C. J. Landers , H. Mallick , D. R. Plichta , M. Prasad , G. Rahnavard , J. Sauk , D. Shungin , Y. Vazquez‐Baeza , R. R. White , J. Braun , L. A. Denson , J. K. Jansson , R. Knight , S. Kugathasan , et al., Nature 2019, 569, 655. 31142855 10.1038/s41586-019-1237-9 PMC6650278

[5] J. Liang , W. Dai , C. Liu , Y. Wen , C. Chen , Y. Xu , S. Huang , S. Hou , C. Li , Y. Chen , W. Wang , H. Tang , Adv. Sci. 2024, 11, 2400206. 10.1002/advs.202400206 PMC11267284 38639442

[6] N. Li , P. Ma , Y. Li , X. Shang , X. Nan , L. Shi , X. Han , J. Liu , Y. Hong , Q. Li , J. Cui , J. Li , G. Peng , Gut Microbes 2023, 15, 2290315. 38062857 10.1080/19490976.2023.2290315 PMC10730201

[7] X. Pan , Q. Zhu , L. L. Pan , J. Sun , Pharmacol. Ther. 2022, 238, 108176. 35346728 10.1016/j.pharmthera.2022.108176

[8] Y. Ma , X. Zhang , B. Xuan , D. Li , N. Yin , L. Ning , Y. L. Zhou , Y. Yan , T. Tong , X. Zhu , X. Huang , M. Hu , Z. Wang , Z. Cui , H. Li , J. Wang , J. Y. Fang , R. Liu , H. Chen , J. Hong , Gut 2024, 73, 268. 37734910 10.1136/gutjnl-2023-330009

[9] L. Fan , L. Yao , Z. Li , Z. Wan , W. Sun , S. Qiu , W. Zhang , D. Xiao , L. Song , G. Yang , Y. Zhang , M. Wei , X. Yang , Adv. Sci. 2023, 10, 2205692. 10.1002/advs.202205692 PMC10190648 36965082

[10] X. Zheng , Q. Jiang , M. Han , F. Ye , M. Wang , Y. Qiu , J. Wang , M. Gao , F. Hou , H. Wang , Cell. Mol. Immunol. 2023, 20, 1367. 37821621 10.1038/s41423-023-01081-2 PMC10616184

[11] J. V. Patankar , C. Becker , Nat. Rev. Gastroenterol. Hepatol. 2020, 17, 543. 32651553 10.1038/s41575-020-0326-4

[12] E. G. Foerster , T. Mukherjee , L. Cabral‐Fernandes , J. Rocha , S. E. Girardin , D. J. Philpott , Autophagy 2022, 18, 86. 33906557 10.1080/15548627.2021.1909406 PMC8865220

[13] S. J. Dixon , J. A. Olzmann , Nat. Rev. Mol. Cell Biol. 2024, 25, 424. 38366038 10.1038/s41580-024-00703-5 PMC12187608

[14] F. Huang , S. Zhang , X. Li , Y. Huang , S. He , L. Luo , Biol. Med. 2022, 188, 375. 10.1016/j.freeradbiomed.2022.06.242 35779691

[15] B. Li , H. Ming , S. Qin , E. C. Nice , J. Dong , Du Z. , C. Huang , Signal Transduction Targeted Ther. 2025, 10, 72. 10.1038/s41392-024-02095-6 PMC11885647 40050273

[16] S. Wang , W. Liu , J. Wang , X. Bai , Life Sci. 2020, 259, 118356. 32861798 10.1016/j.lfs.2020.118356

[17] Y. Yang , L. Li , Q. Hang , Y. Fang , X. Dong , P. Cao , Z. Yin , L. Luo , Redox Biol. 2019, 20, 157. 30326393 10.1016/j.redox.2018.09.019 PMC6197438

[18] O. Ellouze , N. Frikha , S. Ouerghi , T. Mestiri , B. A. M. Salah , Tunis Med. 2011, 89, 738. 22076894

[19] M. H. Zarka , W. J. Bridge , Redox Biol. 2017, 11, 631. 28131081 10.1016/j.redox.2017.01.014 PMC5284489

[20] D. Huang , J. Wang , M. Nie , G. Chen , Y. Zhao , Adv. Mater. 2023, 35, 2301192. 10.1002/adma.202301192 37004147

[21] C. C. Mayorga‐Martinez , M. Fojtu , J. Vyskocil , N. Cho , M. Pumera , Adv. Funct. Mater. 2022, 32, 2207272.

[22] K. Gu , A. Wu , C. Liu , B. Yu , J. He , X. Lai , J. Chen , Y. Luo , H. Yan , P. Zheng , J. Luo , J. Pu , Q. Wang , H. Wang , D. Chen , J. Adv. Res. 2024. 10.1016/j.jare.2024.12.030 PMC12793762 39710300

[23] A. Arranz , C. Doxaki , E. Vergadi , D. L. T. Y. Martinez , K. Vaporidi , E. D. Lagoudaki , E. Ieronymaki , A. Androulidaki , M. Venihaki , A. N. Margioris , E. N. Stathopoulos , P. N. Tsichlis , C. Tsatsanis , Proc. Natl. Acad. Sci. U. S. A. 2012, 109, 9517. 22647600 10.1073/pnas.1119038109 PMC3386059

[24] Y. Liu , X. Luo , Y. Chen , J. Dang , D. Zeng , X. Guo , W. Weng , J. Zhao , X. Shi , J. Chen , B. Dong , S. Zhong , J. Ren , Y. Li , J. Wang , J. Zhang , J. Sun , H. Xu , Y. Lu , D. Brand , S. G. Zheng , Y. Pan , Redox Biol. 2024, 69, 103008. 38142586 10.1016/j.redox.2023.103008 PMC10788633

[25] F. Cao , L. Jin , Y. Gao , Y. Ding , H. Wen , Z. Qian , C. Zhang , L. Hong , H. Yang , J. Zhang , Z. Tong , W. Wang , X. Chen , Z. Mao , Nat. Nanotechnol. 2023, 18, 617. 36973397 10.1038/s41565-023-01346-x

[26] H. Guo , H. Guo , Y. Xie , Y. Chen , C. Lu , Z. Yang , Y. Zhu , Y. Ouyang , Y. Zhang , X. Wang , Redox Biol. 2022, 56, 102441. 35985164 10.1016/j.redox.2022.102441 PMC9411672

[27] Y. Yang , Y. Wang , L. Guo , W. Gao , T. L. Tang , M. Yan , Cell Death Dis. 2022, 13, 355. 35429990 10.1038/s41419-022-04775-z PMC9013379

[28] E. K. Wright , M. A. Kamm , S. M. Teo , M. Inouye , J. Wagner , C. D. Kirkwood , Inflammatory Bowel Dis. 2015, 21, 1219. 10.1097/MIB.0000000000000382 PMC4450900 25844959

[29] C. Yang , I. Mogno , E. J. Contijoch , J. N. Borgerding , V. Aggarwala , Z. Li , S. Siu , E. K. Grasset , D. S. Helmus , M. C. Dubinsky , S. Mehandru , A. Cerutti , J. J. Faith , Cell Host Microbe 2020, 27, 467. 32075742 10.1016/j.chom.2020.01.016 PMC7213796

[30] R. Luo , J. Liu , Q. Cheng , M. Shionoya , C. Gao , R. Wang , Sci. Adv. 2024, 10, ado6798. 10.1126/sciadv.ado6798 PMC11212727 38941458

[31] H. J. Galipeau , E. F. Verdu , Science 2023, 381, 1153. 37708269 10.1126/science.adj9724

[32] J. Li , X. Chen , S. Song , W. Jiang , T. Geng , T. Wang , Y. Xu , Y. Zhu , J. Lu , Y. Xia , R. Wang , Cell Rep. 2025, 44, 115350. 40014451 10.1016/j.celrep.2025.115350

[33] X. Jiang , B. R. Stockwell , M. Conrad , Nat. Rev. Mol. Cell Biol. 2021, 22, 266. 33495651 10.1038/s41580-020-00324-8 PMC8142022

[34] J. Zhang , Y. Zhao , T. Hou , H. Zeng , D. Kalambhe , B. Wang , X. Shen , Y. Huang , J. Controlled Release 2020, 320, 363. 10.1016/j.jconrel.2020.01.047 32001299

[35] D. Jiang , Y. Guo , T. Wang , L. Wang , Y. Yan , L. Xia , R. Bam , Z. Yang , H. Lee , T. Iwawaki , B. Gan , A. C. Koong , Nat. Commun. 2024, 15, 4114. 38750057 10.1038/s41467-024-48330-0 PMC11096184

[36] M. Sylvestre , C. A. Crane , S. H. Pun , Adv. Mater. 2020, 32, 1902007. 10.1002/adma.201902007 PMC7098849 31559665

[37] H. He , Q. Qin , F. Xu , Y. Chen , S. Rao , C. Wang , X. Jiang , X. Lu , C. Xie , Sci. Adv. 2023, 9, adf3887. 10.1126/sciadv.adf3887 PMC10219598 37235662

[38] C. Ma , R. Battat , P. S. Dulai , C. E. Parker , W. J. Sandborn , B. G. Feagan , V. Jairath , Drugs 2019, 79, 1321. 31317509 10.1007/s40265-019-01169-y

[39] D. I. Fudman , R. A. Mcconnell , C. Ha , S. Singh , Clin. Gastroenterol. Hepatol. 2025, 23, 454. 39147217 10.1016/j.cgh.2024.06.050 PMC12180935

[40] D. Ahuja , M. H. Murad , C. Ma , V. Jairath , S. Singh , Am. J. Gastroenterol. 2023, 118, 1618. 36976548 10.14309/ajg.0000000000002263

[41] G. M. Leone , K. Mangano , M. C. Petralia , F. Nicoletti , P. Fagone , J. Clin. Med. 2023, 12, 1630. 36836166 10.3390/jcm12041630 PMC9963154

[42] M. Agrawal , E. S. Kim , J. F. Colombel , J. Crohn's Colitis 2020, 14, S755. 32006031 10.1093/ecco-jcc/jjaa017 PMC7395307

[43] D. Zhao , Y. Li , Z. Zhang , T. Xu , C. Ye , T. Shi , Y. Wang , Mater. Horiz. 2023, 10, 1121. 36637068 10.1039/d2mh01236g

[44] D. Mcmanus , H. J. Novaira , A. Hamers , A. B. Pillai , J. Vis. Exp. 2019, 151, 59821. 10.3791/59821 PMC7759444 31609324

[45] L. Jia , Y. Jiang , L. Wu , J. Fu , Du J. , Z. Luo , L. Guo , J. Xu , Y. Liu , Nat. Commun. 2024, 15, 1617. 38388542 10.1038/s41467-024-45473-y PMC10883948

[46] R. Wang , B. Yan , Y. Yin , X. Wang , M. Wu , T. Wen , Y. Qian , Y. Wang , C. Huang , Y. Zhu , Int. J. Biol. Macromol. 2024, 270, 132441. 38761897 10.1016/j.ijbiomac.2024.132441

