# Supplementary material for: Bioinspired Claw‐Engaged Adhesive Microparticles Armed with γGC Alleviate Ulcerative Colitis via Targeted Suppression of Macrophage Ferroptosis
Source: Adv Sci (Weinh). 2025 Apr 29;12(30):2503903. doi: 10.1002/advs.202503903 (PMC12376637; doi:10.1002/advs.202503903)
Supplement: Supplementary file 1 — Supporting Information [file ADVS-12-2503903-s001.docx]

Supplementary Information for

**Bioinspired Claw-Engaged Adhesive Microparticles Armed with γGC Alleviate Ulcerative Colitis via Targeted Suppression of Macrophage Ferroptosis**

# Rong Wang1,4, †, Jianwei Zhu1,2, †, Jinyi Zhou1, Jinyang Li5, Min Wang5, Yuqi Wu1, Danshan Zhao3, Xiancheng Chen^6^,*, Yuetong Wang3,*, Xiaoyuan Chen2,* , Jianhua Zou2,*

1 Jiangsu Province Key Laboratory for Molecular and Medical Biotechnology, College of Life Science, Nanjing Normal University, Nanjing 210046, Jiangsu, China

2 Departments of Diagnostic Radiology, Surgery, Chemical and Biomolecular Engineering, and Biomedical Engineering, Yong Loo Lin School of Medicine and College of Design and Engineering, National University of Singapore, Singapore 119074, Singapore. Email: chen.shawn@nus.edu.sg, zoujh-93@nus.edu.sg

3 School of Food Science and Pharmaceutical Engineering, Nanjing Normal University, Nanjing 210046, Jiangsu, China. Email: wangyt@nnu.edu.cn

4 Hunan Provincial Key Laboratory of the Research and Development of Novel Pharmaceutical Preparations, Changsha Medical University, Changsha 410219, Hunan, China

5 The First Affiliated Hospital of Nanjing Medical University, Nanjing 210029, Jiangsu, China

6 Department of Critical Care Medicine, Nanjing Drum Tower Hospital, The Affiliated Hospital of Nanjing University Medical School, Nanjing 210029, Jiangsu, China. chenxiancheng-icu@foxmail.com

† These authors contributed equally.


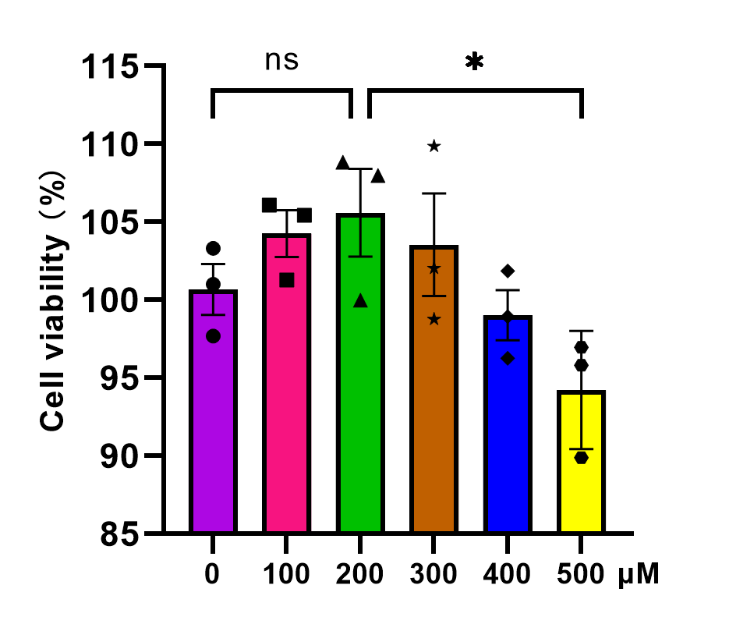


## **Fig. S1.** Cell viability of γGC-treated THP-1 cells. Data are mean ± SEM. **P* < 0.05, ***P* < 0.01, and

****P* < 0.001, ns = no signiﬁcant.


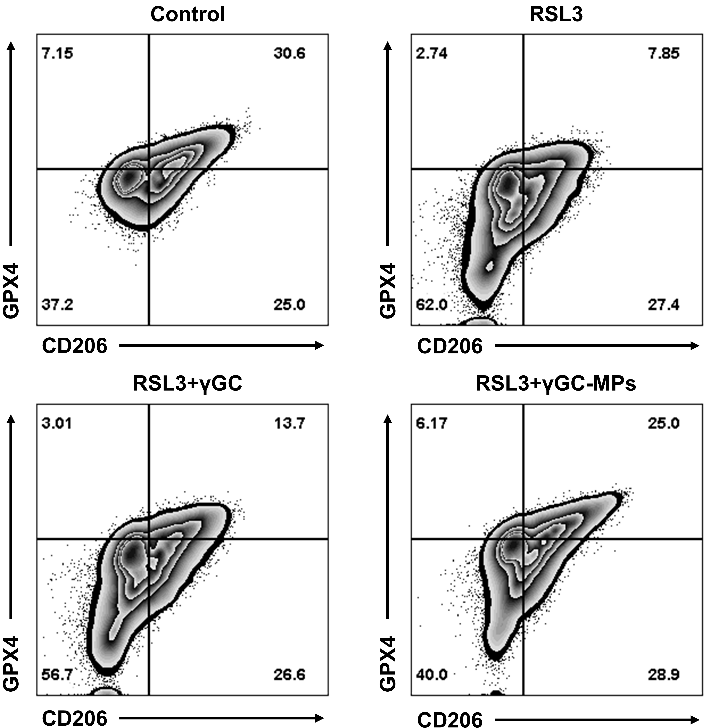


## **Fig. S2.** γGC or γGC-MPs treatment effectively increased the expression of GPX4 and inhibit macrophages M1 polarization after using ferroptosis agonists RSL3.


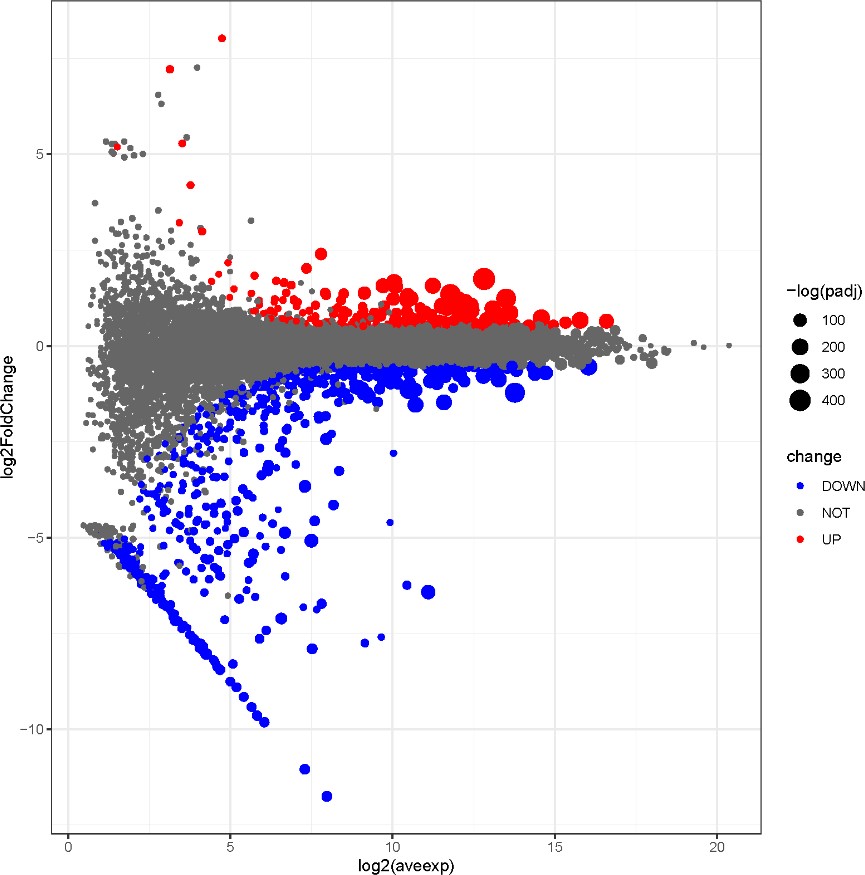


## **Fig. S3.** Differentially expressed genes between the model group (LPS, IFN-γ, and γGC treated) and the

control group (LPS and IFN-γ treated).


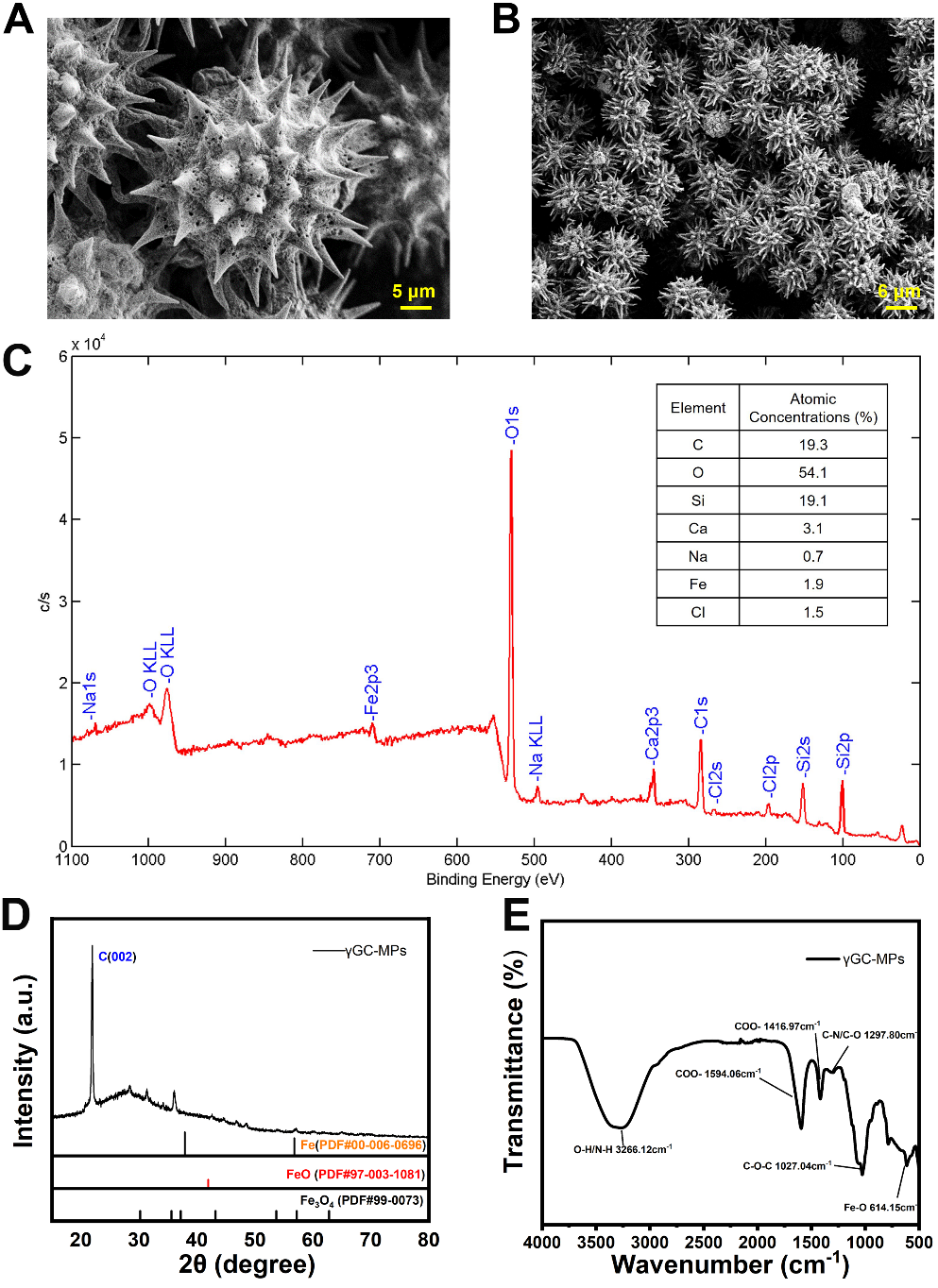


# **Fig. S4.** SEM images of the raw pollen (A) and the CRP@Fe MPs (B). The scale bars are 5 μm and 6 μm, respectively. XPS (C), XRD (D), and FT-IR (E) analysis of γGC-MPs.


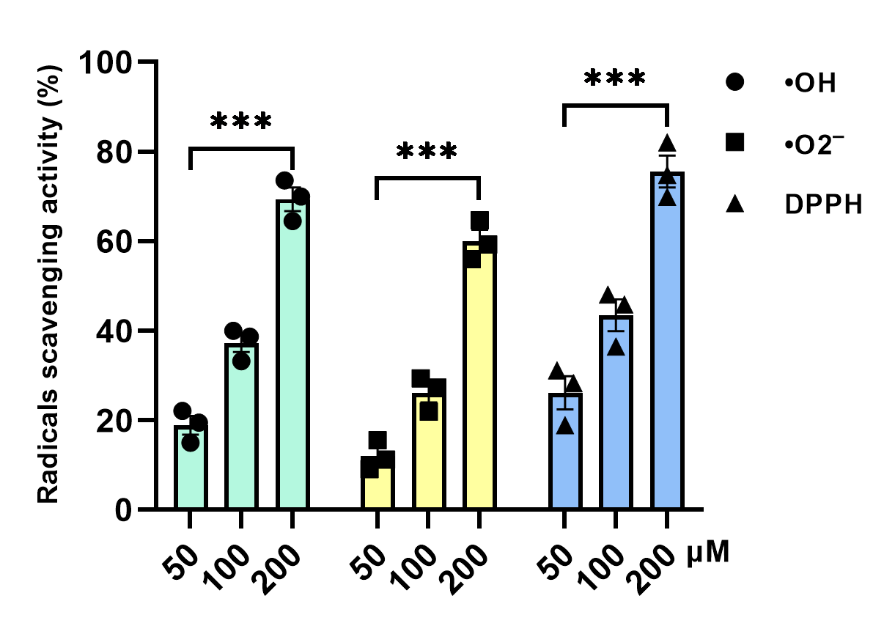


## **Fig. S5.** The •OH, •O2–, and DPPH radical scavenging activities of γGC-MPs. **P* < 0.05, ***P* < 0.01, and

****P* < 0.001, ns = no signiﬁcant.


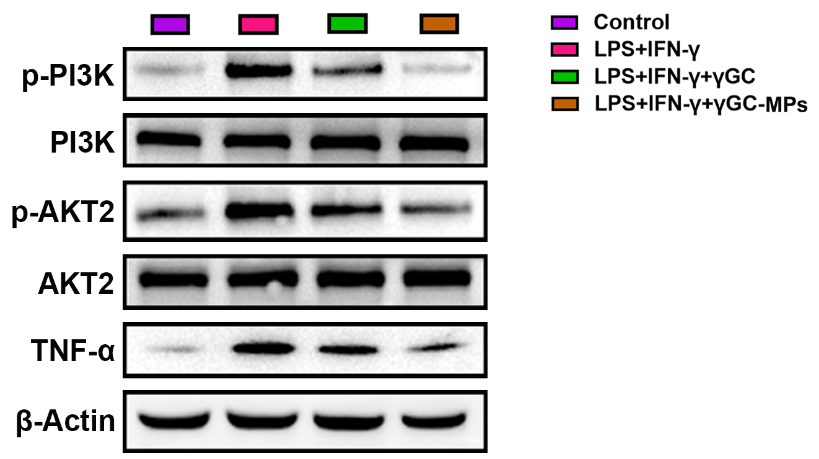


## **Fig. S6.** Protein expressions of p-PI3K, PI3K, p-AKT2, AKT2, and TNF-α after γGC or γGC-MPs treatment were examined by immunoblot.


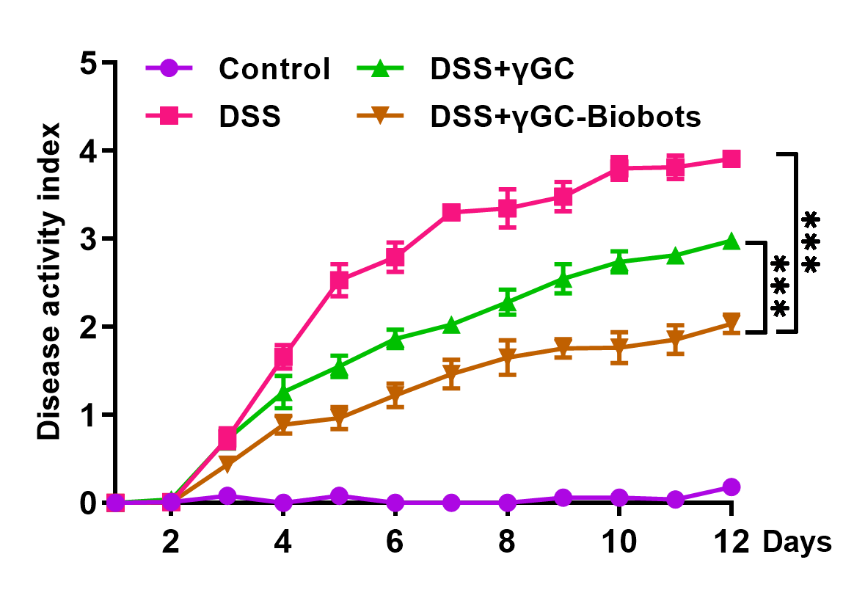


## **Fig. S7.** The DAI line graph for the entire 0-12 day treatment process. **P* < 0.05, ***P* < 0.01, and

****P* < 0.001, ns = no signiﬁcant.


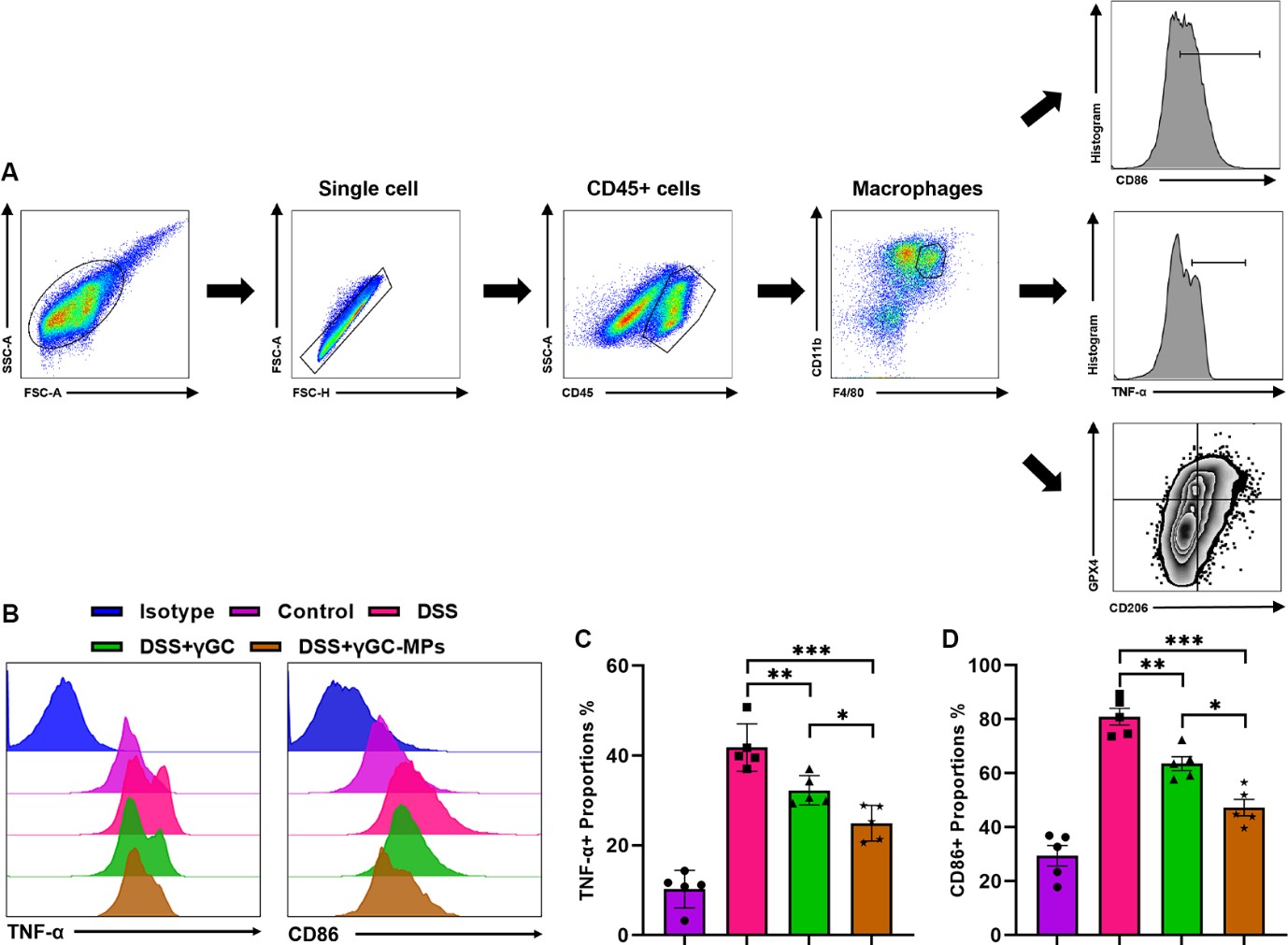


## **Fig. S8.** A. Gating strategies for TNF-α^+^, CD86^+^ and CD206^+^ macrophages (mice intestinal macrophages). B-D. The proportions of TNF-α^+^ and CD86^+^ M1 macrophages were assessed by flow cytometry.


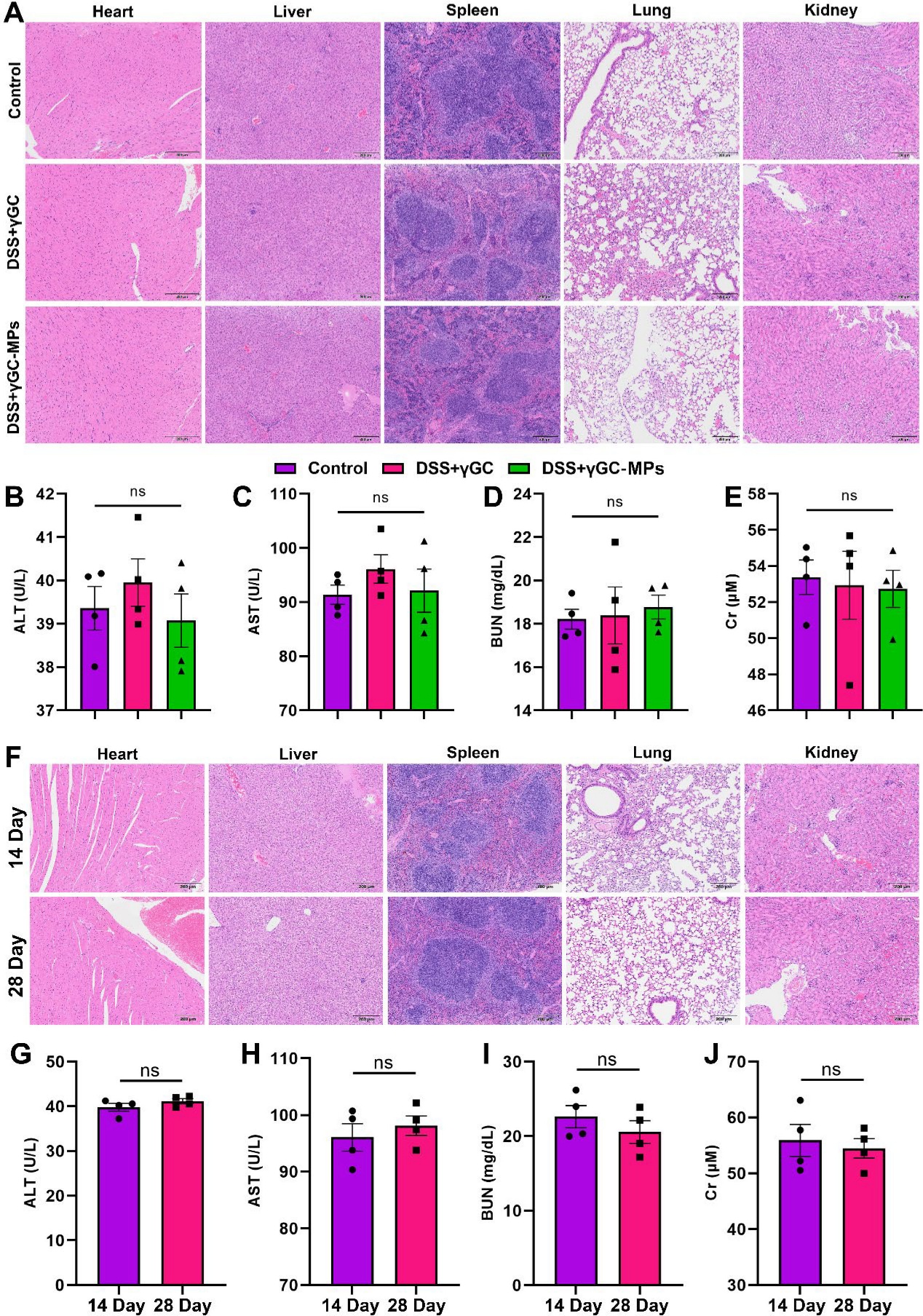


**Fig. S9.** γGC-MPs showed no toxicity *in vivo*. A. H&E staining of sections from five main organs (heart, liver, spleen, lung, and kidney) of mice with different treatments. B-E. Serum indexes, including ALT, AST, BUN, Cr, were tested. F. H&E staining of sections from five main organs (heart, liver, spleen, lung, and kidney) of mice after 14 and 28 days of treatment with γGC-MPs. G-J. Serum indexes, including ALT, AST, BUN, Cr, were tested from mice after 14 and 28 days of treatment with γGC-MPs *P < 0.05, **P < 0.01, and ***P < 0.001, ns = no significant.


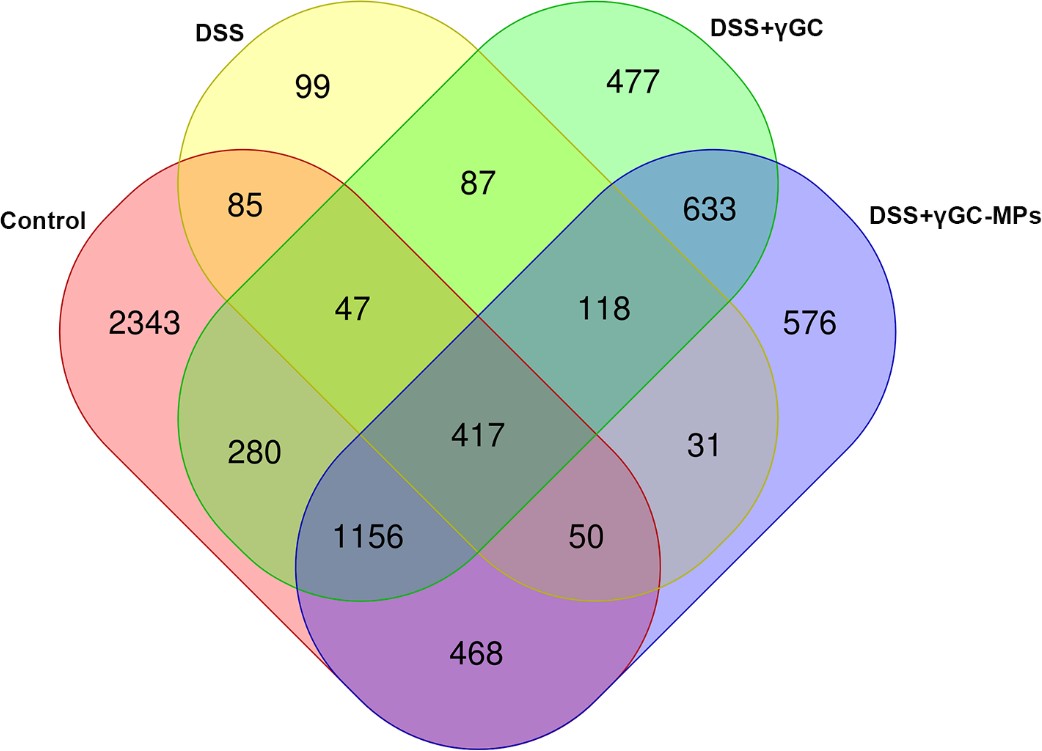


## **Fig. S10.** Venn diagram of species among the four groups (Control, DSS, DSS+γGC, DSS+γGC-MPs).


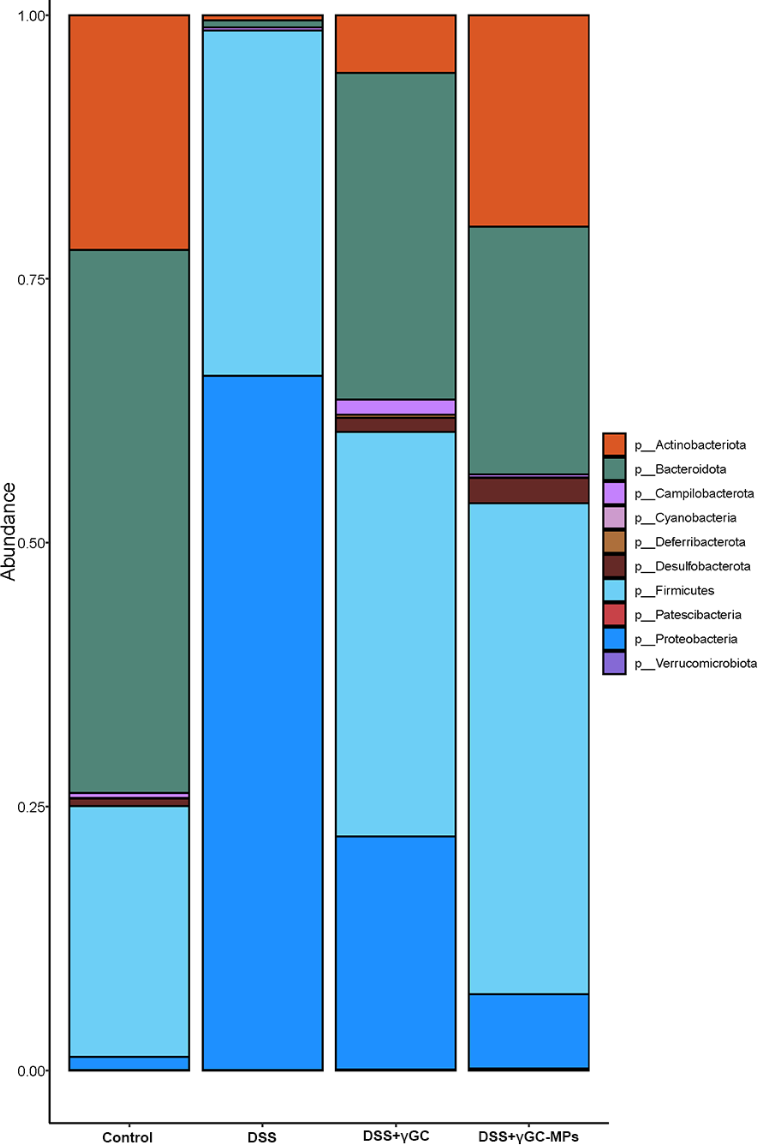


**Fig. S11.** Histogram of phylum abundance among four groups (Control, DSS, DSS+γGC, DSS+γGC- MPs).


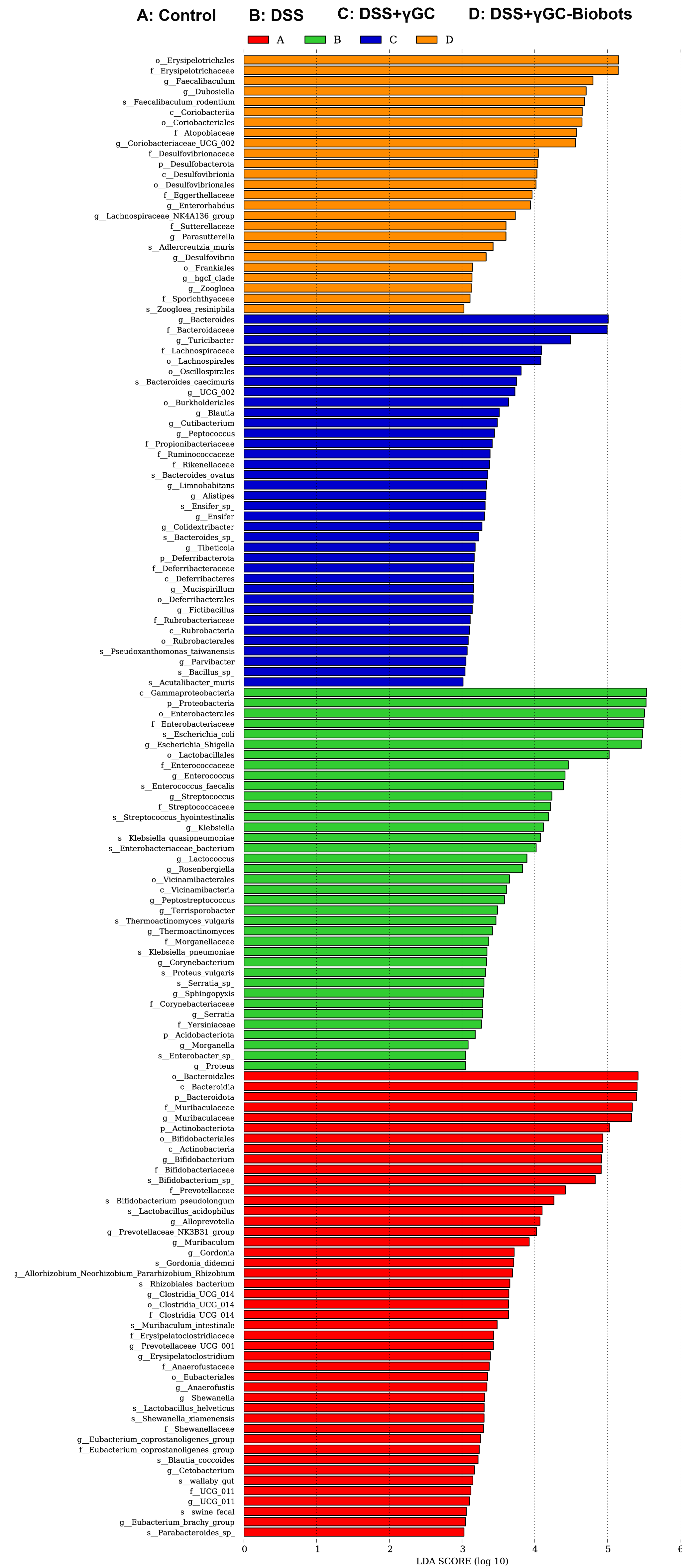


## **Fig. S12.** Taxa listed according to LDA values determined from comparisons between the four groups using the LEfSe method. LDA score > 3.0, *P* < 0.05 indicated a higher relative abundance in the corresponding group than in other groups. A: Control; B: DSS; C: DSS+γGC; D: DSS+γGC-MPs.


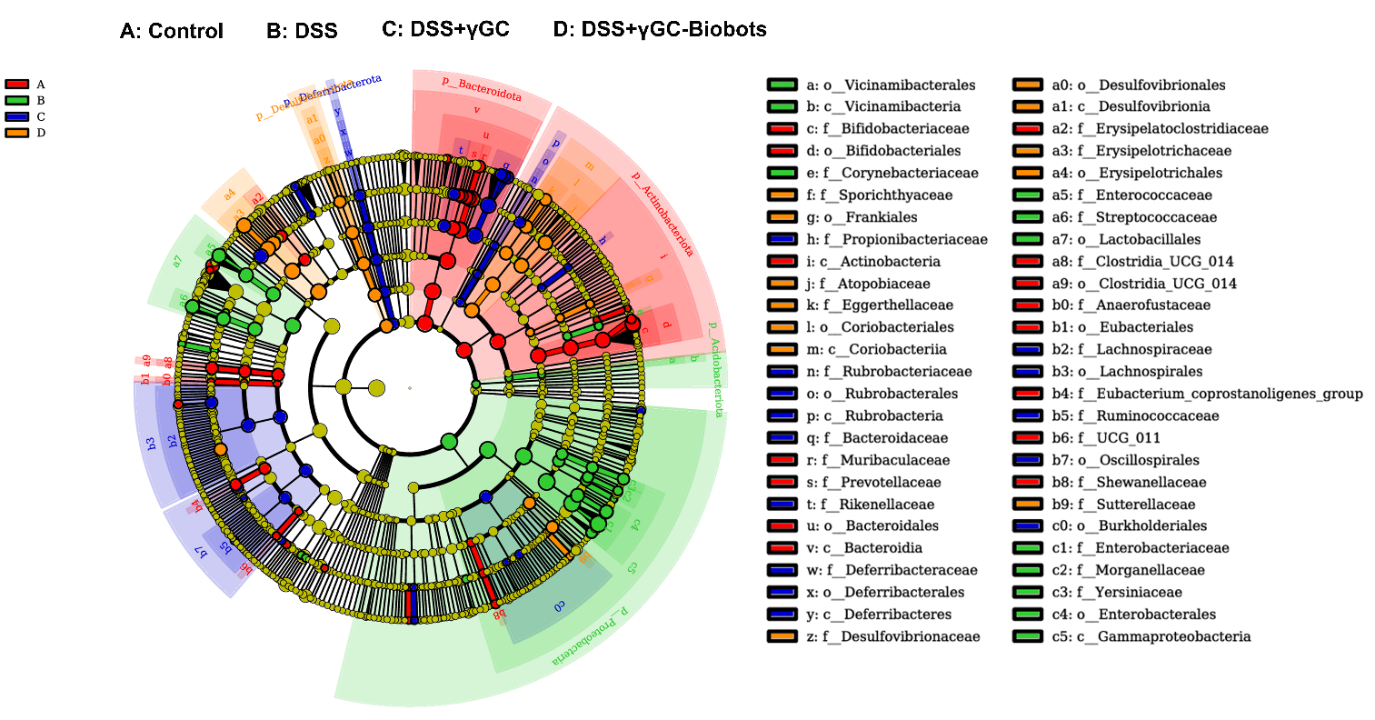


## **Fig. S13.** The cladogram based on LEfSe analysis showed the community composition of the gut microbiota in mice with different treatments. A: Control; B: DSS; C: DSS+γGC; D: DSS+γGC-MPs.

**Supplemental Table 1**

**Baseline characteristics of included UC patients and Healthy volunteers**

|  | **Male gender** | **Age (years)** |
| --- | --- | --- |
| **UC 1** | Female | 53 |
| **UC 2** | Female | 23 |
| **UC 3** | Male | 45 |
| **UC 4** | Female | 52 |
| **UC 5** | Male | 55 |
| **UC 6** | Female | 55 |
| **UC 7** | Female | 53 |
| **UC 8** | Male | 47 |
| **Healthy volunteer 1** | Male | 20 |
| **Healthy volunteer 2** | Male | 55 |
| **Healthy volunteer 3** | Female | 33 |
| **Healthy volunteer 4** | Female | 29 |
| **Healthy volunteer 5** | Female | 34 |
| **Healthy volunteer 6** | Male | 30 |
| **Healthy volunteer 7** | Male | 29 |
| **Healthy volunteer 8** | Male | 32 |

### Supplementary Table 2 Antibodies used in flow cytometry

| **Host/Target Species** | **Antigen/Conjugation** | **Fluorochrome** | **Supplier** | **Cat. No** |
| --- | --- | --- | --- | --- |
| Human | CD45 | BV510 | BioLegend | 368526 |
| Human | CD68 | FITC | BioLegend | 137006 |
| Human | CD206 | AF700 | BioLegend | 321132 |
| Human | CD86 | BV421 | BioLegend | 374212 |
| Human | TNF-α | APC | BioLegend | 502913 |
| Human | CD11b | BV605 | BioLegend | 124218 |
| Mouse | CD45 | BV510 | BioLegend | 103138 |
| Mouse | CD86 | BV421 | BioLegend | 105031 |
| Mouse | CD206 | AF700 | BioLegend | 141734 |
| Mouse | CD11b | BV605 | BioLegend | 356518 |
| Mouse | F4/80 | FITC | BioLegend | 123108 |
| Mouse | TNF-α | APC | BioLegend | 506307 |
| Mouse | GPX4 | \ | Thermo Fisher | PA5-109274 |
| Rabbit | PE | \ | BioLegend | 406421 |

#### Abbreviations: PE, phycoerythrin; FITC, fluorescein isothiocyanate; APC, allophycocyanin; BV, Brilliant Violet; AF, Alexa Fluor.

**Supplementary Table 3**

**Antibodies used in IF and ICC**

| **Host/Target Species** | **Antigen/Conjugation** | **Supplier** | **Cat. No** | **Dilution** | **Application** |
| --- | --- | --- | --- | --- | --- |
| Human, Mouse | CD86 | NOVUS | NBP2-25208 | 1:100 | IF, ICC |
| Human, Mouse | GPX4 | Abcam | ab125066 | 1:400 | IF, ICC |
| Mouse | TNF-α | Abcam | ab307164 | 1:100 | IF, ICC |
| Mouse | p-AKT2 | Affinity | AF3264 | 1:100 | IF, ICC |
| Mouse | ZO-1 | Abcam | ab307799 | 1:400 | IF |
| Mouse | Occludin | Abcam | ab216327 | 1:400 | IF |
| Mouse | Claudin-1 | Abcam | ab307692 | 1:400 | IF |
| Rabbit | HRP | Abcam | Ab6721 | 1:1000 | IHC |
| Mouse | HRP | Abcam | Ab205719 | 1:2000 | IHC |
| Rabbit | CY5 | Abcam | Ab6564 | 1:500 | IF |
| Rabbit | AF488 | Abcam | Ab150077 | 1:500 | ICC |
| Mouse | AF594 | Abcam | Ab150116 | 1:500 | IF |
| Rat | AF488 | Abcam | Ab150157 | 1:1000 | IF |

Abbreviations: IF, immunofluorescence; ICC, immunocytochemistry; AF, Alexa Fluor; HRP, horseradish peroxidase.

### Supplementary Table 4 Primary antibodies used in WB

| **Host/Target Species** | **Antigen/Conjugation** | **Supplier** | **Cat. No** | **Dilution** | **Application** |
| --- | --- | --- | --- | --- | --- |
| Human, Mouse | GPX4 | Abcam | ab125066 | 1:1000 | WB, IF |
| Human, Mouse | TNF-α | Abcam | ab307164 | 1:1000 | WB, IF |
| Human, Mouse | β-Actin | Abcam | mAbcam8226 | 1:3000 | WB, IF |
| Human | Nrf2 | Abways | CY5136 | 1:1000 | WB |
| Human, Mouse | p-PI3K | HUABIO | HA721672 | 1:1000 | WB |
| Human, Mouse | PI3K | HUABIO | EM1701-62 | 1:1000 | WB |
| Human, Mouse | p-AKT2 | Affinity | AF3264 | 1:1000 | WB, IF |
| Human, Mouse | AKT2 | HUABIO | RT1027 | 1:100 | WB, IF |
|  | Goat anti-Rabbit IgG  (H+L)-HRP | Bioworld | BS13278 | 1:40000 | WB |
|  | Goat anti-Mouse IgG  (H+L)-HRP | Bioworld | BS12478 | 1:40000 | WB |

#### Abbreviations: IF, immunofluorescence; WB, western blot; HRP, horseradish peroxidase.

**Supplementary Table 5**

| **Species** | **Gene** | **Forward Sequence (5’-3’)** | **Reverse Sequence (5’-3’)** |
| --- | --- | --- | --- |
| Human | Arg1 | TCATCTGGGTGGATGCTCACAC | GAGAATCCTGGCACATCGGGAA |
| Human | CD206 | AGCCAACACCAGCTCCTCAAGA | CAAAACGCTCGCGCATTGTCCA |
| Human | iNOS | GCTCTACACCTCCAATGTGACC | CTGCCGAGATTTGAGCCTCATG |
| Human | TNF-α | CTCTTCTGCCTGCTGCACTTTG | ATGGGCTACAGGCTTGTCACTC |
| Human | β-Actin | CACCATTGGCAATGAGCGGTTC | AGGTCTTTGCGGATGTCCACGT |

**qRT-PCR primer sequences**

**Supplementary Table 6**

**Particle size distribution**

| **Radius of MP (μm)** | **Relative Frequency** | | **Radius of CRP@Fe (μm)** | **Relative Frequency** |
| --- | --- | --- | --- | --- |
| 35 | | 0.11 | 7 | 0.07 |
| 36 | | 0.18 | 7.5 | 0.1 |
| 37 | | 0.18 | 8 | 0.11 |
| 38 | | 0.25 | 8.5 | 0.29 |
| 39 | | 0.16 | 9 | 0.16 |
| 40 | | 0.08 | 9.5 | 0.18 |
| 41 | | 0.02 | 10 | 0.08 |
| 42 | | 0.02 | 10.5 | 0.01 |
